# A Novel Virus Causes Scale Drop Disease in *Lates calcarifer*

**DOI:** 10.1371/journal.ppat.1005074

**Published:** 2015-08-07

**Authors:** Ad de Groof, Lars Guelen, Martin Deijs, Yorick van der Wal, Masato Miyata, Kah Sing Ng, Lotte van Grinsven, Bartjan Simmelink, Yvonne Biermann, Luc Grisez, Jan van Lent, Anthony de Ronde, Siow Foong Chang, Carla Schrier, Lia van der Hoek

**Affiliations:** 1 MSD Animal Health / Intervet International bv., Department Discovery & Technology, Boxmeer, The Netherlands; 2 Laboratory of Experimental Virology, Department of Medical Microbiology, Center for Infection and Immunity Amsterdam (CINIMA), Academic Medical Center, University of Amsterdam, Amsterdam, The Netherlands; 3 MSD Animal Health Innovation Pte Ltd, Singapore; 4 Laboratory of Virology and Wageningen Electron Microscopy Centre, Wageningen University, Wageningen, The Netherlands; Cleveland Clinic Foundation, UNITED STATES

## Abstract

From 1992 onwards, outbreaks of a previously unknown illness have been reported in Asian seabass (*Lates calcarifer*) kept in maricultures in Southeast Asia. The most striking symptom of this emerging disease is the loss of scales. It was referred to as scale drop syndrome, but the etiology remained enigmatic. By using a next-generation virus discovery technique, VIDISCA-454, sequences of an unknown virus were detected in serum of diseased fish. The near complete genome sequence of the virus was determined, which shows a unique genome organization, and low levels of identity to known members of the *Iridoviridae*. Based on homology of a series of putatively encoded proteins, the virus is a novel member of the *Megalocytivirus* genus of the *Iridoviridae* family. The virus was isolated and propagated in cell culture, where it caused a cytopathogenic effect in infected Asian seabass kidney and brain cells. Electron microscopy revealed icosahedral virions of about 140 nm, characteristic for the *Iridoviridae*. *In vitro* cultured virus induced scale drop syndrome in Asian seabass *in vivo* and the virus could be reisolated from these infected fish. These findings show that the virus is the causative agent for the scale drop syndrome, as each of Koch’s postulates is fulfilled. We have named the virus Scale Drop Disease Virus. Vaccines prepared from BEI- and formalin inactivated virus, as well as from *E*. *coli* produced major capsid protein provide efficacious protection against scale drop disease.

## Author Summary

Asian seabass or *Lates calcarifer* is a large, valuable fish kept in maricultures. Scale drop syndrome is an emerging disease in this species that currently results in significant economic losses for affected farms. Mortality rates can become as high as 50%, both in juvenile and adult fish. With the increasing mariculture of *L*. *calcarifer*, it is expected that economic losses due to the syndrome will increase as well. We provide conclusive evidence that the single causative agent for the syndrome is a newly identified member of the genus *Megalocytivirus* that has been designated scale drop disease virus.

## Introduction

Scale drop syndrome in *Lates calcarifer*, Asian seabass, was first reported in 1992 in Penang, Malaysia, and since then outbreaks have been seen in Indonesia and in the Strait of Malacca. The phenotypic symptoms and pathology of the syndrome were described in detail by Gibson-Kueh et al. [1]. Typically, affected fish are characterized by darkened bodies, scale loss, tail and fin erosion, pallor of gills, and sometimes exophthalmia. Furthermore, fish often show lethargic behavior and severely affected fish stop schooling, sometimes show spiral swimming and a large proportion of the fish eventually die. The pathological symptoms comprise vasculitis in all major organs, including the skin and the brain, tissue degeneration, hemorrhages and necrosis. The cumulative mortality is estimated around 40–50%. The syndrome is so far described to affect Asian seabass only, both juvenile and adult fish, and seems to follow a seasonal pattern: the south-west monsoon/inter-monsoon season starting around September may be a trigger. It is an illness with unknown etiology of which the incidence is on the rise in commercial fish farms. As Asian seabass is a large, valuable fish kept in maricultures, scale drop syndrome currently results in significant economic losses for affected farms. With the increasing aquaculture of *L*. *calcarifer*, from 11,000 tonnes in 1990 to 75,000 tonnes in 2012 [2], the syndrome is expected to occur with increased frequency. The identification of the cause of the disease, and if possible, the development of a vaccine, are therefore highly desired.

Scale drop syndrome spreads between cages, indicating that an infectious agent is involved. Initially, it was believed that scale drop syndrome was caused by infections with *Tenacibaculum maritimum*, but so far this or other known microorganisms could not be linked to scale drop syndrome [1]. Since antibiotic treatment does not work to prevent disease, we hypothesized that a yet unknown viral agent is involved.

Detection of an unknown virus requires specialized techniques. Nowadays, next generation sequencing platforms combined with viral purification and library preparations provide and excellent method to identify viruses of which the genome composition is unknown. The VIDISCA method (Virus discovery cDNA-AFLP) is one of the library preparation methods which has been successfully used to identify several novel viruses [3–7]. Our aim was to detect a possible viral pathogen using this approach.

In this study the VIDISCA library of sera of fish affected by scale drop syndrome was sequenced via Roche-454 next generation sequencing, enabling identification and characterization of a novel virus, which we named Scale Drop Disease Virus (SDDV). Three candidate vaccines were designed and all Koch's postulates were fulfilled.

## Results

### Viral sequences identified in *Lates calcarifer* with scale drop syndrome

Four serum samples of scale drop syndrome-affected fish from Singapore were analyzed with VIDISCA-454. A total of 42,918 sequence reads were obtained of which 15 showed limited identity to known viruses in GenBank, indicating that the samples may contain an unknown virus. At the nucleotide level, these sequence reads showed identity to conserved parts of the megalocytiviruses from the family *Iridoviridae*.

A real time qPCR was developed on a VIDISCA fragment which appeared to be part of the gene encoding the putative DNA-dependent RNA polymerase of the virus. Three out of four serum samples of the affected fish were PCR positive. Organ material from spleen, heart and kidney of diseased fish was also PCR positive. All serum, spleen, and kidney control samples from healthy fish remained negative in the qPCR. Testing of 30 more sera from early and late stage scale drop syndrome affected fish collected at a mariculture farm in Indonesia revealed another 25 positive fish, whereas none of 6 healthy control fish samples were positive (S1 Table).

The near complete genome sequence of the novel virus was obtained via genome walking. The genome length is at least 124,244 bp, consists of dsDNA, and it contains no less than 129 ORFs. A Blastn search showed that the closest relatives are among the megalocytiviruses, however, the identity was low (depending on the gene, at most 60%). Dot plot analyses confirmed that there are large differences with all known members of the *Iridoviridae*, including the megalocytiviruses (S1 Fig). In addition, an unusual low % G+C is present in the viral genome: only 37% whereas this value ranges between 53% and 55% for the other full length sequenced megalocytiviruses [8].

**Fig 1:**
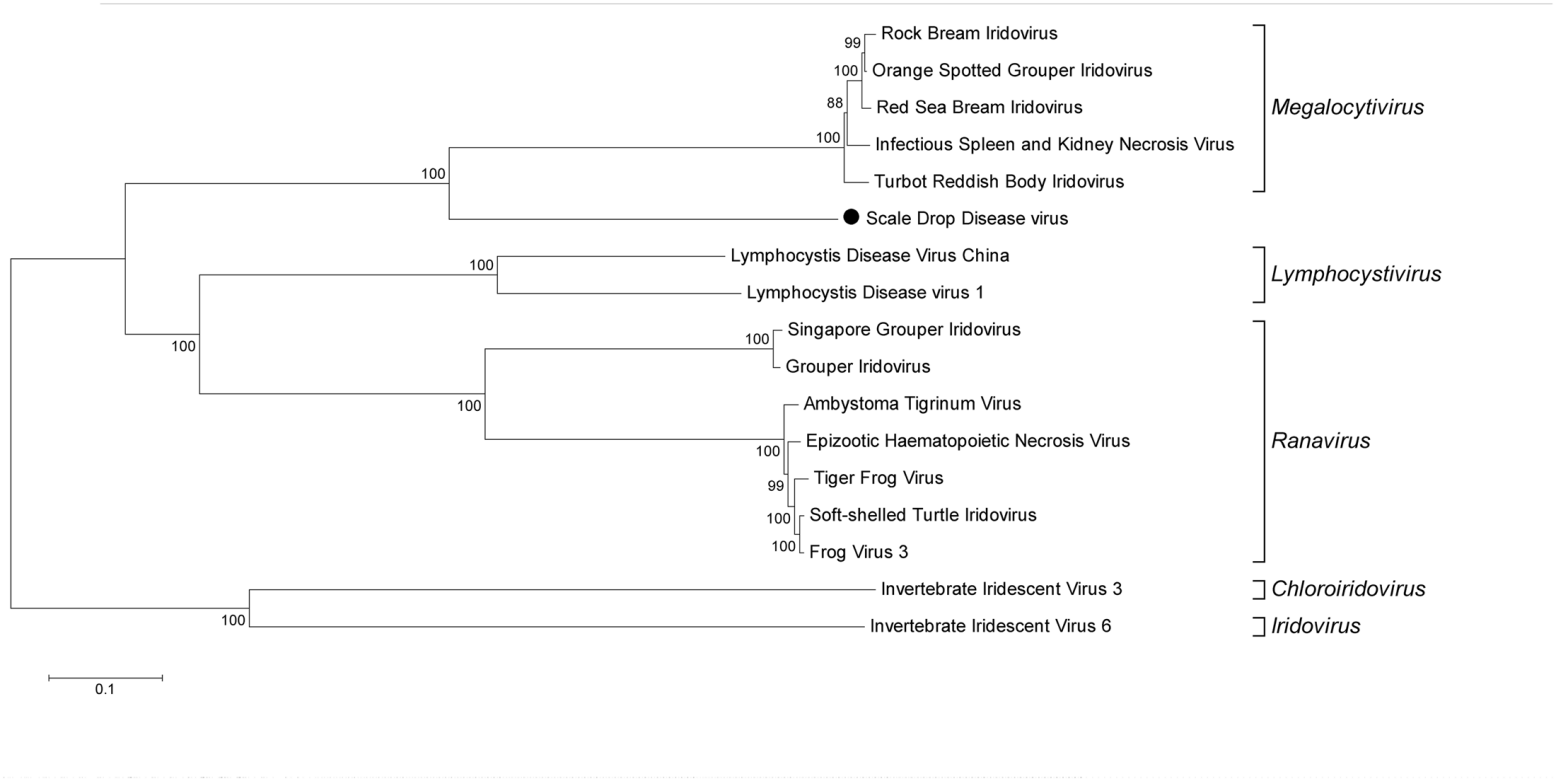
Phylogenetic clustering of SDDV within the *Iridoviridae* family. Deduced amino acid sequences of 26 conserved genes (see S2 Table) were aligned with the corresponding genes of *Iridoviridae* of which the full length genome sequence is available. The genes of each virus are placed in the same order and the total was used for phylogenetic analysis, similar as described by Eaton et al. [9]. The neighbor-joining method with pairwise deletion within the MEGA-5 package was used, bootstrap values (for 500 replications) are provided at the root of the clusters and the scale bar is a measure of the proportion of divergence. SDDV is indicated with • in the tree.

At this point it was not clear whether the novel virus was the causative agent or an innocent bystander of scale drop syndrome, but as a working hypothesis we called this virus Scale Drop Disease Virus (SDDV). In literature, a set of 26 conserved *Iridoviridae* genes is used for genome comparison [9] and all of these 26 are present in the near complete sequence of SDDV. The location of each gene was determined and compared to the locations in other known members of the *Iridoviridae* family for which the full genome has been determined. S2 Table shows that the position of the conserved genes is unique and distinctively different from the other viruses. Phylogenetic analysis based on the deduced amino acid sequences of the 26 conserved proteins revealed that the virus clusters with the megalocytiviruses, yet it does not cluster as tightly as the other members (Fig 1). Full genome sequences are however not available for all known megalocytiviruses, therefore an additional phylogenetic analysis was performed using the deduced amino acid sequences of the major capsid protein (MCP), which allowed inclusion of all (sequenced) members of the *Iridoviridae* family. S2 Fig shows that there are no close relatives, none of the known viruses clusters close to SDDV.

### Virus culture

**Fig 2:**
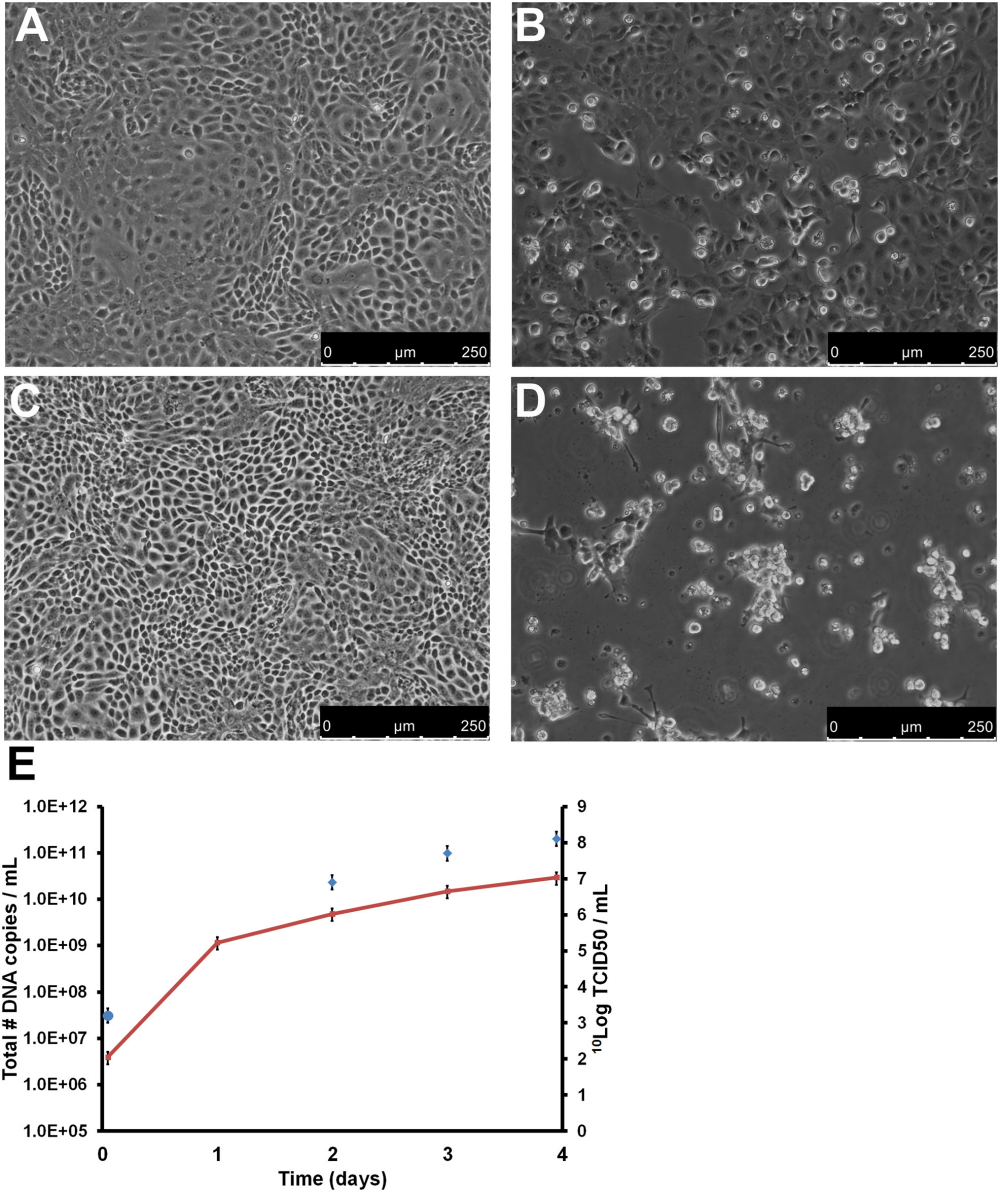
Replication of SDDV on SK21 cells. SK21 cells were inoculated with an MOI of 0.01 TCID_50_/cell SDDV (passage 3). (A/C) Negative control confluent monolayer of SK21 cells on day 3 (A) and 4 (C); (B/D) SDDV-infected SK21 cells on day 3 (B) and 4 (D) after inoculation; (E) SDDV genome copy number/mL (red line and left y-axis) and infectious titer (blue diamonds and right y-axis) of the infected SK21 cells in time. Error bars represent the standard deviation.

PCR-positive sera were pooled and used to inoculate seabass kidney (SK) SK21 cells. A negative control was composed of the pooled sera from three PCR-negative fish without clinical signs of scale drop syndrome. Enlarged cells were already observed two days after inoculation of the SK21 cell cultures, and after six days, a high level of cytopathogenic effect (CPE) was observed. Cultures were harvested by applying three freeze-thaw cycles, and replication of the virus was confirmed by qPCR on the harvest. Subsequently, the harvest was used for a second passage on SK21 cells using 10 DNA copies per cell as inoculum, which corresponds to a multiplicity of infection (MOI) of 0.01 TCID_50_/cell (determined by back calculation). CPE was clearly visible in the second passage on day four and it was complete (100%) on day five. A third passage was started using the day four harvest of the second passage, again using 10 DNA copies per cell (MOI 0.01 TCID_50_/cell) as inoculum. The CPE was evident in the infected cultures, defined initially by distinct morphological changes in the cells, and at later times this was coupled with an increase in detachment from the culture vessel (shown in Fig 2B and 2D). Viral replication was monitored by determining the number of viral genome copies on day 1 to 4 and the infectious titer on day 2, 3 and 4. The genome copy number increased from 2 x 10^6^ to 3 x 10^10^ genome copies/mL. The virus titer (TCID_50_), increased from 3.2 ^10^log TCID_50_/mL to 8.1 ^10^log TCID_50_/mL on day 4 (Fig 2E). Similar results were obtained when the virus was inoculated on Asian seabass brain cells (SBB, established at MSD AH, S. Koumans, D. Remmers, S3 Fig). Negative control inoculation did not induce CPE and remained qPCR-negative during three subsequent passages on SK21 and SBB cells.

The possibility that CPE was caused by dilution of a toxic factor instead of a virus was excluded by conducting serial passages of the SK21 culture harvest. A total of 5 successive passages at MOI 0.01 TCID_50_/cell were conducted on SK21 cells in T25 flasks. Cells were harvested at the time of near-complete CPE, usually day 3–4 after inoculation. Harvests of the cultures were titrated and showed to be between 7.6–8.2 ^10^Log TCID_50_/mL culture harvest. Contamination by viral or bacterial contaminants like mycoplasmas was excluded as well by using the tests as described in the European Pharmacopeia [10]. Furthermore VIDISCA-454 was performed on the culture supernatant of passage 3. Besides the novel virus, no sequences with significant identity to any virus were detected.

To confirm correlation between presence of the virus and the induction of CPE, a qPCR analysis of DNA samples isolated from CPE-positive and -negative wells in a representative plate of the titration assay was performed. In all wells that were CPE-positive a high concentration of SDDV DNA was detected (> 10^8^ copies/mL, shown in S3 Table).

### Electron microscopy visualization of SDDV

**Fig 3:**
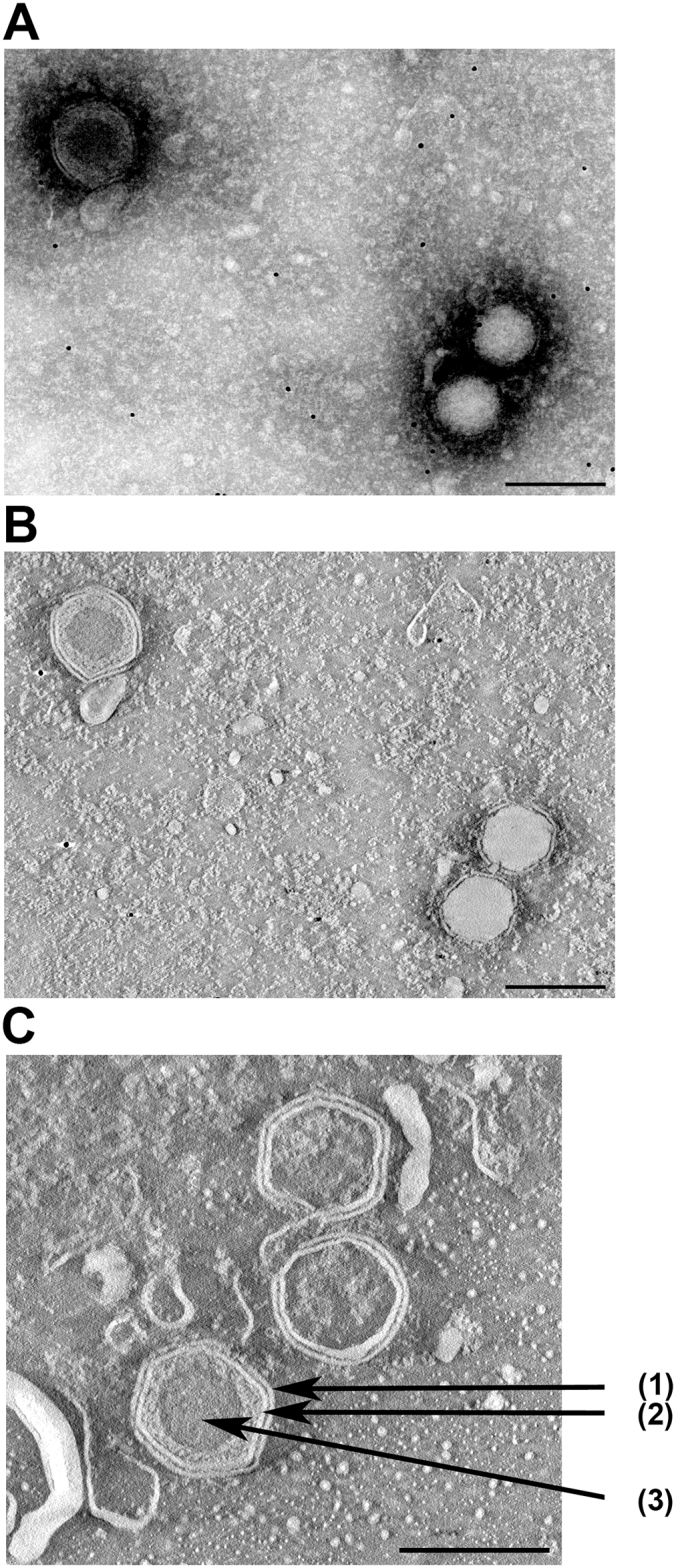
Electron microscopy of SDDV. Hexagon particles with (upper left) or without (lower right) outer membrane as visualized by by negative staining (A) followed by tomography (B). In (C) the outer membrane (1), capsid (2) and inner core (3) of a different enveloped particle are indicated by arrows. In (B) and (C) (ortho)slices through virus particles are shown.

Members of the *Iridoviridae* have an outer protein capsid composed of capsomers, covering an inner lipid membrane bilayer that envelopes the genome. The lipid membrane adopts an icosahedral morphology that roughly follows the contour defined by the outer layer of capsomers [11]. Electron microscopy of SDDV shows virions with a diameter of about 140 nm (Fig 3). In the concentrated virus suspension two types of particles can be discerned: particles that have the outer membrane and capsid, where the core appears dark and particles that have lost the outer membrane and appear with a lighter-colored core (Fig 3A). Most particles had no or had lost the outer membrane. The icosahedral symmetry, the inner core and capsid are typical for members of the *Iridoviridae*. An internal membrane is not visible (Fig 3).

### Chloroform treatment

As *Iridoviridae* family members have been reported to be present as enveloped and non-enveloped viruses, we investigated if a lipid envelope was essential for infectivity of the virus in SK21 cells by applying chloroform treatment. Titrations of the virus harvest incubated with 0%, 10%, and 50% (v/v) chloroform yielded different TCID_50_ values. The ^10^log TCID_50_/mL of the 0% (v/v) control was 7.5, compared to 5.5 of the sample treated with 10% (v/v) chloroform and 5.4 ^10^log TCID_50_/mL of the 50% (v/v) chloroform-treated sample S4 Fig). This indicates that at least a subset of virions remained infectious after chloroform treatment. The 100-fold decrease in TCID_50_ could be the result of loss of the enveloped subset of virions that are sensitive to chloroform, but this could also be an effect of chloroform on naked virions. A lipid envelope, if present, does not seem essential for infectivity of the virus.

### Infection of cultured virus induces scale drop syndrome in *Lates calcarifer*

**Fig 4:**
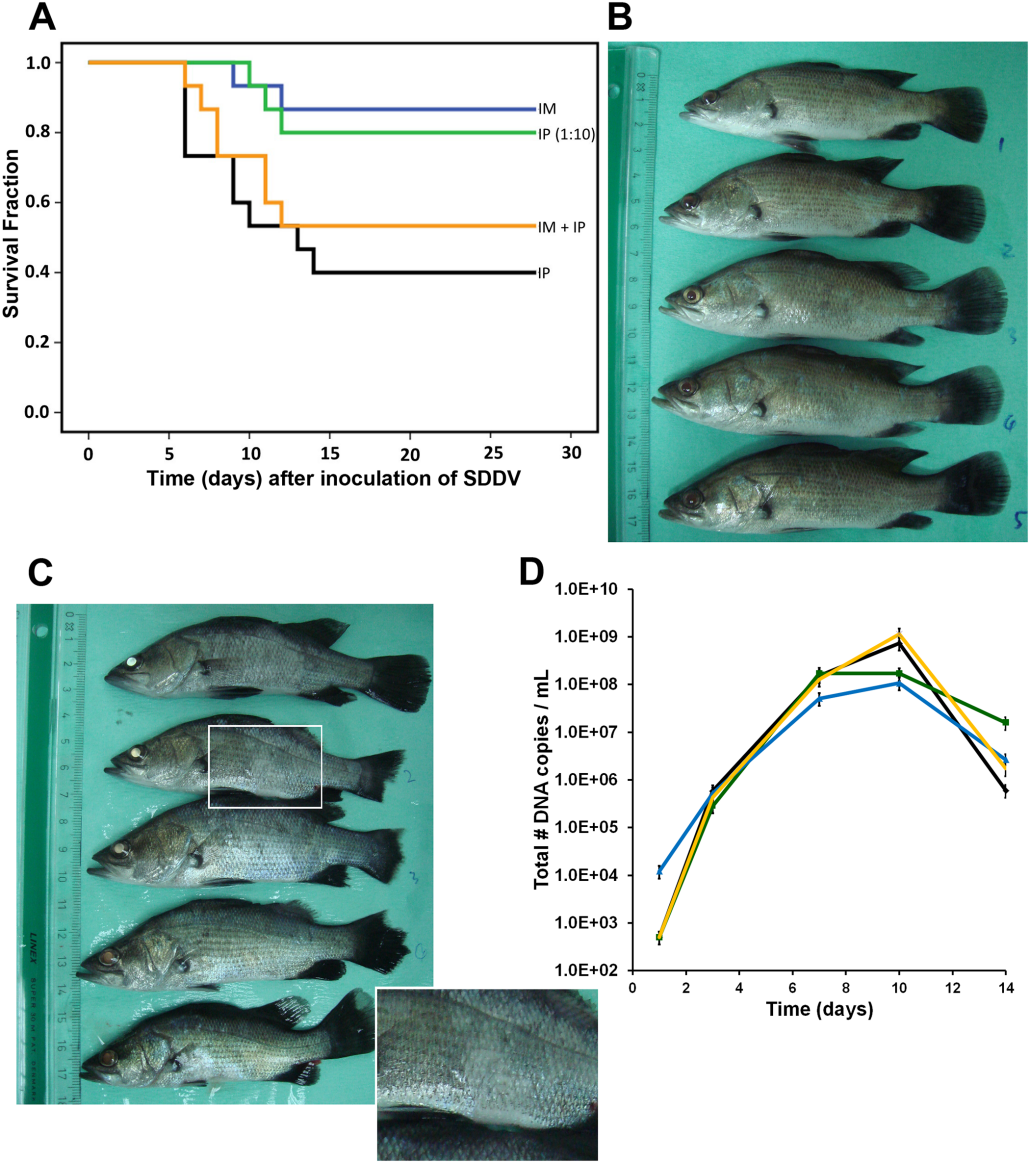
Inoculation of SDDV in *Lates calcarifer*. (A) Kaplan-Meier survival curve of infected fish with 5x10^6^ TCID_50_/fish (IP—black line) or 5.5 x10^5^ TCID_50_/fish (IM—blue line) or both (IP + IM—yellow line). IP (1:10; green line) dose equaled 5.5 x10^5^ TCID_50_/fish. (B) Control fish at day 10; (C) SDDV-infected fish (IP-high dose) at day 10 after infection. Note the fin erosion, tail erosion, body color variations, white or less mucus (inset), and changes in eye color. (D) SDDV genome copy number in serum of the fish on day 1, 7, 10, and 14 after infection. Diamond—black line: IP (mortality 60%); Triangle—blue line: IM (mortality 13%); Single cross—yellow line: IP + IM (mortality 47%); Square—green line: IP 1:10 (mortality 20%).

To examine if the newly discovered virus was indeed the causative agent of scale drop syndrome, *L*. *calcarifer* were infected with virus culture harvest. Virus culture harvests (passage 3) were applied via different routes of infection: intraperitoneal (IP; 0.1 mL or 5.5 x 10^6^ TCID_50_/fish), intramuscular (IM; 0.01 mL or 5.5 x 10^5^ TCID_50_/fish), and a combination (IP 0.1 mL + IM 0.01 mL). A fourth group of fish was infected IP with 0.1 mL of a 1:10 dilution of culture harvest in virus dilution buffer (PBS with increased salt concentration (1.5% NaCL); IP 1:10) to match the infectivity dose in the IM infection. The fifth group contained control fish. Records of fish mortalities in the observation tanks between day 0 and day 28 are shown in Fig 4A (15 fish/group). Clinical symptoms characteristic of scale drop syndrome, including tail and fin erosion, broken fins, scale loss and exophthalmia, were clearly visible on day 7–14 after infection in the IP-challenged group (Fig 4C; day 10) and the IP- + IM-challenged group. In the IP-challenged fish, mortality was observed from 5 days post-infection. Cumulative mortality reached 60% for the IP group and 47% for the IP + IM group. Fish that received an IP 1:10-dose only or an IM dose showed less severe clinical symptoms and a later onset of mortality and lower cumulative mortality: 20% (IP 1:10) and 13% (IM). No mortality was observed in control fish. Pooled serum samples collected from animals at 1, 3, 7, 10 and 14 days after infection were analyzed by qPCR for the presence of viral DNA. Viral DNA was detected in all groups on day 3, 7, 10 and 14, and not in the control group (Fig 4D). The amount of viral DNA copies peaked around 10 days post-infection.

### Reisolation and culture of SDDV from inoculated diseased fish

Further fulfillment of Koch’s postulates [12] was achieved by confirming the presence of the infectious agent in the experimentally infected fish. Pooled sera of day 7 and 10 of the abovementioned infections were used to inoculate SK21 cells. All sera were tested at 1:100 and 1:1000 dilution. Sera from all infected groups gave CPE on day 3 with the exception of the 1:1000 IP 1:10 dose day 10 serum, which gave CPE on day 7 (S4 Table). Control sera remained CPE negative in this assay. The presence of SDDV in the CPE positive cultures was confirmed by PCR and sequencing of the PCR products.

### Three prototype vaccines show efficacious protection against scale drop syndrome

A vaccination-challenge study was performed to investigate whether a vaccine could protect the fish from scale drop syndrome. The optimal SDDV challenge dose for IP injection in 67 g fish was determined to define the best virus concentration to infect fish following vaccination. Groups of 15 fish were injected with 2.0 x 10^8^ TCID^50^/fish, 2.0 x 10^7^ TCID_50_/fish, and 2.0 x 10^6^ TCID_50_/fish using a 0.1 mL IP injection. Mortality reached 100% (15/15) in the 2.0 x 10^7^ TCID_50_/fish and 2.0 x 10^6^ TCID_50_/fish groups, but unexpectedly not in the group that received the highest viral dose (2.0 x 10^8^ TCID_50_/fish: 11/15 or 73%) at day 27 post inoculation. Based on these results a dose of 2.0 x 10^7^ TCID_50_/fish was chosen to challenge the fish after vaccination.

**Fig 5:**
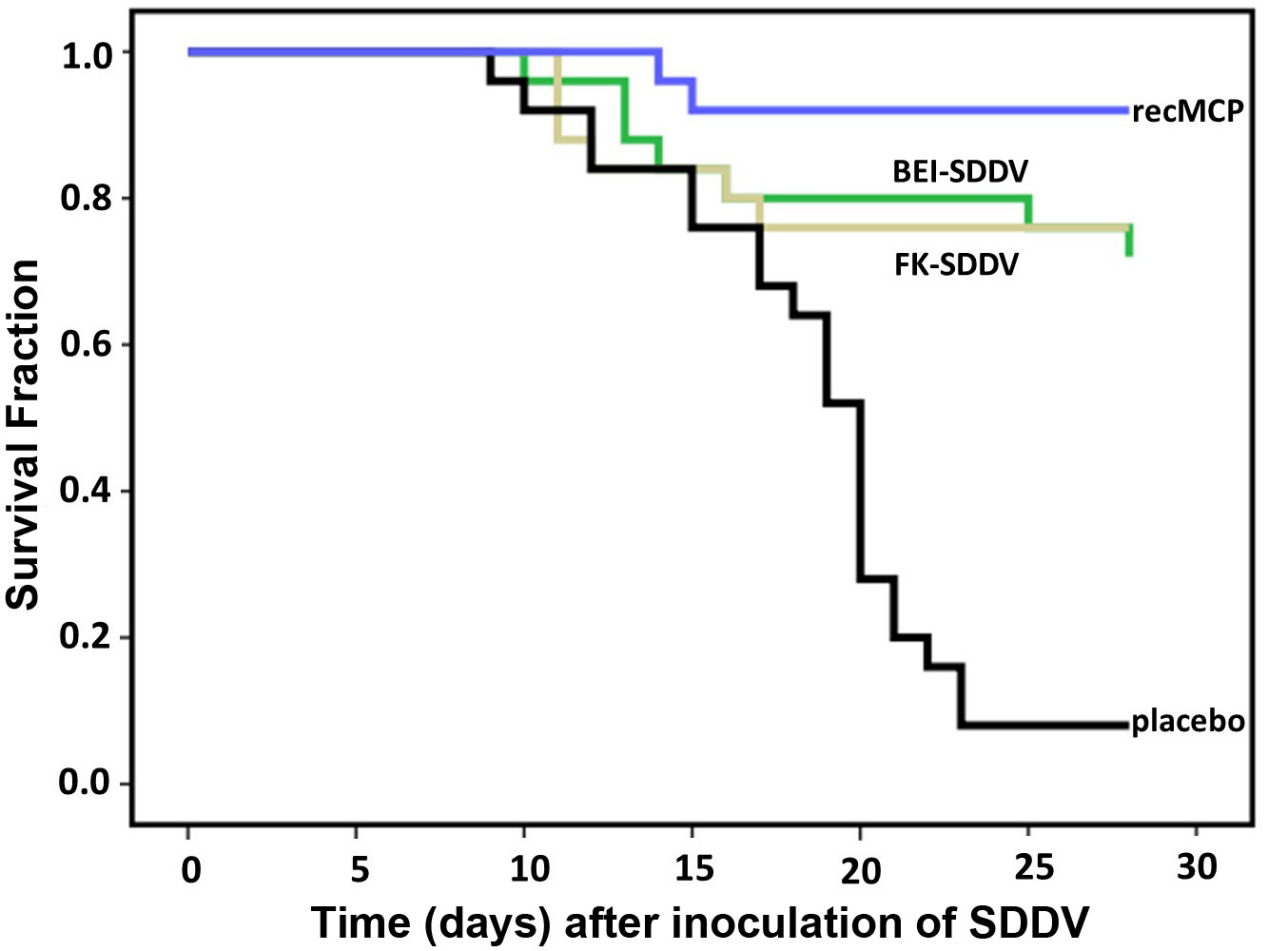
Protection of scale drop syndrome by vaccination of *Lates calcarifer*. Kaplan-Meier survival curve of fish vaccinated with formalin-inactivated virus (FK-SDDV), binary ethyleneimine-inactivated virus (BEI-SDDV), recombinant MCP protein (recMCP), or diluent control vaccine (placebo). Twenty-eight days after vaccination the fish were challenged with 2 x 10^7^ TCID_50_ SDDV per fish (intraperitoneal), mortality was scored until day 28 after challenge. All three vaccines provided >70% RPS against disease (p<0.001, Tarone-Ware test).

A formalin-inactivated virus vaccine, a BEI (binary ethyleneimine)-inactivated virus vaccine, and a recombinant MCP protein produced in E.coli (recMCP) vaccine were tested. An oil-adjuvanted vaccine made with dilution buffer only was used as control. Vaccines were administered as 0.1 mL IP dose at day 0 of the experiment when the fish averaged a weight of 60 g. Fish were challenged with an IP challenge of 2.0 x 10^7^ TCID_50_/fish on day 28 post vaccination, when the average weight had reached 83 g. Survival after challenge was monitored for 28 days (Fig 5). Mortality in the control group was high with only 8% survival after 28 days (2/25), whereas all three prototype vaccines provided protection with relative protection percentages of 74% (formalin-inactivated), 70% (BEI-inactivated) and 91% (recMCP). The virus concentrations of the formalin-inactivated vaccine and the BEI-inactivated vaccine differed 10-fold (Table 1 and Fig 5). To examine whether the formalin-inactivated vaccine could provide better protection at a higher dose, a formalin-inactivated vaccine was also made from the same virus stock (10^8^ TCID_50_/mL) that was used to make the BEI-inactivated vaccine. The relative protection percentage of this vaccine was however equal (70%).

**Table 1:** Vaccines.

Vaccine^a^	Antigen	Diluent	Concentration^b^
FK-SDDV	Culture harvest, inactivated	Culture medium	1 x 10^7^ TCID_50_/mL
BEI-SDDV	Culture harvest, inactivated	Culture medium	1 x 10^8^ TCID_50_/mL
recMCP-SDDV	GST-his-SDDV-MCP	PBS	27.2 μg/mL
Placebo	None	PBS	N.A.

^a^ FK-SDDV: formalin-inactivated virus, BEI-SDDV: binary ethyleneimine-inactivated virus, recMCP-SDDV: recombinant MCP protein.

^b^ 0.1 mL vaccine administered per fish (IP), concentration is prior to inactivation for inactivated vaccines. N.A.: Not applicable.

The presence of viral DNA was examined in sera of 5 surviving vaccinated-challenged fish on day 28 post infection, of each vaccine group, and the two surviving fish of the placebo vaccination. The two sera of the surviving placebo vaccinated fish were both positive for SDDV DNA. Also 3 of the 5 sera of the fish receiving the BEI-inactivated SDDV vaccine were positive for SDDV DNA, indicating that residual replication of the virus occurred. The sera of the formalin-inactivated and recMCP vaccinated fish were all negative for SDDV DNA (S5 Table).

The Kaplan Meier survival curve of fish vaccinated with placebo was significantly different from those of the vaccinated fish (p<0.001, Tarone-Ware test), thus all three vaccines provide protection against death caused by SDDV infection. The recMCP had the highest relative protection percentage (91%), which may suggest that this vaccine provides a better protection than inactivated virus, however statistical tests showed that the difference between the protection by recMCP and the inactivated virus vaccines was not significant. The commercial MSD vaccine Aquavac IridoV against red seabream iridovirus (RSIV) showed no cross protection against SDDV (47% survival in placebo-vaccinated and 47% survival in Aquavac IridoV-vaccinated fish).

## Discussion

This study describes the characterization of a virus which infects *Lates calcarifer* and causes scale drop syndrome. This previously unknown virus belongs to the *Iridoviridae* family and has only limited similarity with the known members. The pathogen was detected exclusively in sera and organs from fish with scale drop syndrome. To establish its role in scale drop syndrome, the virus was cultured and the culture harvests were used to infect *Lates calcarifer*, which resulted in the development of scale drop syndrome. The virus could subsequently be reisolated from the affected fish; hence all of Koch’s postulates were fulfilled. We propose to name the virus Scale Drop Disease virus (SDDV), as we provide conclusive evidence that the virus is the single causative agent for the syndrome that can now be classified as scale drop disease.

SDDV is a member of the *Iridoviridae*, a family of viruses that consists of five genera. The three genera that infect vertebrates (fish, amphibians and reptiles) are *Ranavirus*, *Lymphocystivirus* and *Megalocytivirus*. The two genera that infect invertebrates (insects, crustaceans and possibly mollusks) are *Chloriridovirus* and *Iridovirus*. Analyses of putatively encoded proteins of conserved genes of SDDV revealed most phylogenetic relatedness with the megalocytiviruses. SDDV clusters with the megalocytiviruses but forms a separate branch within this genus. The megalocytiviruses are well known pathogens in tropical fish in South East Asia and Japan. One of the most extensively investigated megalocytiviruses is RSIV. RSIV is the causative agent of a disease with high mortalities that was first found in red sea bream (*Pagrus major*), which is a species of major importance for the tropical fish industry [13]. Later, two other megalocytiviruses that cause systemic diseases in fish, infectious spleen and kidney necrosis virus (ISKNV) and turbot reddish body iridovirus (TRBIV) were discovered [14]. Based on sequence analysis and serological studies, it has been concluded by the ICTV that the group of ISKNV/TRBIV/RSIV are strains of the same viral species. Because the full genome of ISKNV was the first to be determined, ISKNV is the type species of this group [15]. Only threespine stickleback virus and SDDV cluster apart from ISKNV (see S2 Fig).

The genome size and genome type of SDDV are characteristic for the *Iridoviridae*. Their genome is a single molecule of linear, double stranded DNA between 105 and 212 kbp, which is terminally redundant and circularly permuted [9,16]. Also based on its morphology, the classification of SDDV as a member of the *Iridoviridae* is justified (Fig 3). Irodovirids display icosahedral symmetry and are characterized by a central DNA-protein complex, an outer proteinaceous capsid and an intermediate lipid membrane associated with polypeptides that covers and protects the genetic material. The diameter of SDDV, 140 nm, is in the range of 120–200 nm that has been reported for other the *Iridoviridae* family [14]. The virions can be both enveloped and non-enveloped, depending on the mode of exit from the cells, i.e. through lysis or budding. The EM pictures revealed that only some virus particles contained an envelope (Fig 3), but one should keep in mind that loss of an envelope can also be caused by the experimental procedures used for EM. Nevertheless, the remaining infectivity after chloroform treatment shows that an envelope is not essential for SDDV infectivity. The EM pictures did not reveal whether SDDV has an internal lipid membrane. Advanced cryo-electron microscopy will be necessary to elucidate if such membrane is present or absent.

An efficacious protection against SDDV would be very beneficial for the industry. Formalin-inactivated and BEI-inactivated SDDV whole virus vaccines established in this study show promising protection, although these vaccines have to be further developed. Previous studies on RSIV have shown that inactivated whole virus vaccines offer good protection against disease [17,18]. An efficacious commercial formalin-inactivated vaccine against RSIV is available from MSD (Aquavac IridoV, an oil-adjuvanted vaccine). We tested if the Aquavac IridoV vaccine provides cross-protection against SDDV, but such cross-protection could not be shown. Most likely, SDDV and RSIV do not have sufficient antigenic epitopes in common, which is not unexpected based on the fact that the genetic differences and biological mechanisms of replication differ considerably between the viruses. The RSIV vaccine from Biken [19], which is a formalin-inactivated RSIV culture supernatant of GF cells, was not tested.

In addition to the inactivated whole virus vaccine, the vaccine based on a recMCP protein produced in *E*.*coli* showed highly efficacious protection against SDDV. It has already been suggested for other members of the *Iridoviridae* that MCP and other viral surface proteins are candidates for vaccine development, but so far no highly efficacious subunit vaccine has been described. Caipaing et al. [20] showed that only fish vaccinated with inactivated intact RSIV virus and not fish vaccinated with protein components such as MCP survived RSIV challenge. Fu et al. [21] showed that recombinant ISKNV MCP from prokaryotic origin emulsified with ISA 763 oil at a dose of 50 μg/fish gave a relative percent survival (RPS) of 64.3%. Our vaccination challenge study with 27 μg/mL (2.7 μg/fish) SDDV recMCP from prokaryotic origin in ISA 763A VG oil provided a RPS of 91%, and thus the SDDV-MCP protein vaccine is a very promising candidate for vaccine development against SDDV. Further studies are necessary to investigate what factors determine the immune response against recombinant viral proteins, in particular MCP, in the different megalocytiviruses.

Apart from the clear significance to all stakeholders involved in Asian mariculture, we provide data that contribute to the complete picture of the pathogenicity of the *Iridoviridae*. This is not only valuable to all researchers that investigate this family of viruses, but also the broader community, e.g. ecologists, oceanographers etc, that may encounter unsuspected and/or emerging diseases in fish.

## Materials and Methods

### Fish samples

*L*. *calcarifer* samples from Singapore were collected at a mariculture farm in December 2010 and July 2011 from fish with typical signs of scale drop syndrome. Serum was collected from blood that was allowed to clot for 2 hours at room temperature and subsequently stored overnight at 4°C. Blood was centrifuged and serum was transferred to tubes and stored at < -20°C. Various organs from diseased fish were collected (heart, spleen, kidney). From healthy fish, two spleens, two anterior kidneys, two dorsal kidneys and two serum samples were obtained. Three groups of fish from an Indonesian farm were collected in June and November 2012: six control fish from a cage with no symptoms of scale drop syndrome, twenty-five fish from cages with early stage scale drop syndrome and five fish from a cage at the peak mortality stage of scale drop syndrome. The total list of samples is supplied in the S1 Table.

### Ethics statement

All animal procedures were carried out in Singapore, and performed in strict accordance with the specific regulations that govern animal research in Singapore, following the Guidelines set forth by the National Advisory Committee for Laboratory Animal Research (NACLAR) on the Proper Care and Use of Animals for Scientific Purposes (2004). Research on animals is regulated by the Agri-Food and Veterinary Authority of Singapore under the Animal and Birds Act, Animals and Birds (Care and Use of Animals for Scientific Purposes) Rules. The site carrying out the research is audited annually and licensed to perform animal research (License No. VR001). The MSD AH Innovation Ltd IACUC reviewed and approved the animal care and use protocol (license number EXT-EXP/05082011).

### VIDISCA-454

Four serum samples of affected fish and two serum samples of healthy appearing fish from Singapore were analyzed by VIDISCA-454. The VIDISCA-454 was performed as described by de Vries et al. [22]. In short, serum was centrifuged for 10 minutes at 10,000 x *g* and the supernatant was treated with TURBO DNase (2U/μl, Ambion). Subsequently, nucleic acids were extracted by the Boom extraction method [23]. A reverse transcription reaction with Superscript II (Invitrogen) was performed using non-ribosomal random hexamers [24]. Subsequently, second strand DNA synthesis was performed with 5 U of Klenow fragment (New England Biolabs). Double-stranded DNA was purified by phenol/chloroform extraction and ethanol precipitation and digested with Mse I restriction enzyme (New England Biolabs). Adaptors with different Multiplex Identifier sequences (MIDs) were ligated to the digested fragments of the different samples. Before PCR amplification, the fragments were purified with AMPure XP beads (Agencourt AMPure XP PCR, Beckman Coulter). Next, a 28 cycles PCR with adaptor-annealing primers was performed. The program of the PCR-reaction was: 5 min 95°C, and cycles of 1 min 95°C, 1 min 55°C, and 2 min 72°C, followed by 10 min 72°C and 10 min 4°C. After purification with AMPure XP beads, the purified DNA was quantified with the Quant-it dsDNA HS Qubit kit (Invitrogen) and diluted to 10^7^ copies/μl. Samples were pooled and Kapa PCR (Kapa Biosystems) was performed to determine the quantity of amplifiable DNA in each pool. Subsequently, the Bioanalyser (hsDNA chip, Agencourt) was used to determine the average nucleotide length of the libraries. The pools were diluted until 10^6^ copies/μl, titrated with beads (DNA:beads ratio of 0.5:1, 1:1, 2:1 and 4:1) and used in an emulsion PCR according to the supplier’s protocol (LIB-A SV emPCR kit). Sequencing was done on a 2 region GS FLX Titanium PicoTiterPlate (70x75) with the GS FLX Titanium XLR 70 Sequencing kit (Roche). Sequence reads were analyzed using the blastn and blastp algorithms (National Center for Biotechnology Information).

### DNA isolation for qPCR

Tissue samples (spleen, kidney and heart) were homogenized to a 10% (w/v) homogenate in PBS using glass beads. DNA was isolated from homogenized tissue samples, serum and tissue culture virus harvest using the Qiagen DNeasy Blood & Tissue kit according to the manufacturer’s instructions with some adjustments: Fifty μL of tissue homogenate was digested by Proteinase K (20 μl, 600 mAU/ml, Qiagen), mixed with 130 μL solution ATL (Qiagen), and incubated for 60 minutes at room temperature. Fifty μL of serum was mixed with 20 μl Proteinase K and 150 μL PBS, or 200 μl cell culture harvests was added to 20 μL Proteinase K. The mixtures were subsequently incubated with 20 μL RNase A (20 mg/mL) left for 2 minutes at room temperature and from this step on the manufacturer’s instructions were followed.

### Virus quantitation

Sequences from the putative DNA dependent RNA polymerase gene (beta subunit) were used to design primers for PCR analysis. A primer set for red sea bream iridovirus (RSIV) was used as a control (S6 Table). A quantitative PCR was set up based on a 164 bp amplicon of the putative DNA dependent RNA polymerase. The qPCR reactions were performed on a Bio-Rad CFX thermocycler and contained 1 U SuperTaq (HT Biotechnology Ltd.), 1x qPCR buffer (0.5 M KCl, 0.1 M Tris-HCl), 300 nM dNTPs (HT Biotechnology Ltd.), 200 nM forward primer, 200 nM reverse primer, 300 nM probe, 3.5 mM MgCl_2_ and 2 μl template DNA in a total volume of 25 μl. See S6 Table for detailed primer and probe information. Cycling conditions were 95°C for 4 min, followed by 35 cycles at 95°C for 30 sec, 50°C for 30 sec and 72°C for 30 sec. The rampspeed was set to 1.5°C/sec from 95°C to 50°C, and from 50°C to 72°C. Data were analyzed using Bio-Rad CFX Manager 2.0 software. A duplicate measurement of a dilution series of a cloned PCR product in pCR4-TOPO (Invitrogen) functioned as a standard curve. Positive or negative classification of the samples was based on the threshold cycle, as compared to the standard curve. The specificity of the qPCR was checked by gel electrophoresis of the amplified PCR product. Calibration curves with slope and y intercept were calculated by the CFX software, and PCR efficiency calculated from the slope was between 95% and 105%. The r^2^ of the calibration curve was >0.99. The lower detection limit of the qPCR is 50 copies/μL.

### Genome walking

The viral sequences identified after performing VIDISCA-454 were used as template for primer design to perform gap-filling PCRs on DNA isolated from serum. Furthermore, DNA libraries digested with either Csp6I, CviAII or AseI were used to sequence fragments that overlap with sequences obtained with VIDISCA. The gap-filling PCR products and the overlapping PCR products were sequenced using BigDye terminator chemistry (BigDye Terminator v1.1 Cycle Sequencing Kit, Applied Biosystems). Sequences were analyzed using Codoncode Aligner Software (version 4.0.4). Open reading frames (ORFs) were identified via ORF finder [25]. Only ORFs larger than 300 nt were scored, with the exception of the ORF putatively encoding the transcription elongation factor TFIIS which is smaller than 300 nt. The near full length sequence is deposited in GENBANK under accession number KR139659.

### BLASTN, dotplot, and phylogenetic tree construction

Blastn (“somewhat familiar sequences”) searches were performed to identify the closest relative of SDDV, and the similarity in genome organization via dot plot analysis. The following parameters were used: match/mismatch scores: 1,-1 and gap costs: existence 2, extension 1 [26]. Nucleotide and protein sequence alignments were generated using the multiple sequence alignment tool ClustalW. Phylogenetic trees were created with MEGA5 software using the neighbor-joining method, with partial deletion in case of gaps or insertions [27]. Only DNA sequences encoding continuous ORFs were included in an alignment. A bootstrap analysis of 500 replicates was performed to provide confidences to the clustering.

### Virus culture

The Seabass kidney SK21 cell line was established from *Lates calcarifer* kidney by the Schweitzer Biotech Company (Taiwan). The cell line was cloned at MSD Animal Health in order to obtain a line that supports virus replication optimally (S. Koumans, MSD Animal Health). SK21 cells were cultured in 899 mL Leibovitz's L15 medium (Life Technologies) supplemented with 100 mL (10% (v/v)) FCS (Biochrom AG) and 1 mL (1% (v/v)) of a Neomycin Polymyxin antibiotics solution (1000 x stock) at 28°C in a humidified incubator at ambient CO_2_ levels. For virus culture, cells were seeded at 3.0 x 10^4^ cells/cm^2^ and cultured for 24 hours prior to inoculation. The monolayer had reached a cell density of 3.5–4.0 x 10^4^ cells/cm^2^ at the time of infection.

Serum of early scale drop syndrome fish from Indonesia was used for virus culture. The initial inoculum consisted of a 1:10 (v/v) dilution of this pooled serum in culture medium. Culture medium was removed from the flask and the monolayer (3.5–4.0 x 10^4^ cells/cm^2^) was covered with inoculum at 28°C and ambient CO_2_ levels for a minimum of 30 min. The inoculum was removed, fresh culture medium was added and cells were cultured until CPE was observed using an inverted light microscope (Olympus CKX41). Virus was harvested by freeze-thawing (-70°C to 4°C; once to three times), and subsequently the harvest was cleared from cell debris by centrifugation at 1000 x *g* for 5 minutes at 4°C. The virus was passaged by inoculating subconfluent monolayers of cells (3.0 x 10^4^ cells/cm^2^), as described above, with a freeze-thawed and cleared harvest of a previous passage, which was diluted in culture medium to a concentration of 0.01 TCID_50_/cell.

### Virus titration on SK21 cells

An SK21 cell suspension (6.0 x 10^4^ cells/mL in cold (4°C) 50% culture medium + 50% Leibovitz's L15 medium (Life Technologies)) was seeded at 100 μL/well on a 96 wells tissue culture microtiter plate. The plates were incubated for 24 hours at 28°C in a humid atmosphere at ambient CO_2_ levels, where cells reached a density of 3.5–4.0 x 10^4^ cells/cm^2^ at the time of inoculation. Ten-fold serial dilutions of cell culture harvest or serum were prepared up to 10^−9^ dilution and 100 μl per well was added to the SK21 cells. Per dilution, 10 wells were inoculated and negative controls were included in each experiment. The plates were incubated at 28°C for 9–10 days and screened for CPE with an inverted light microscope. TCID_50_ values were determined according to the method and calculations described by Reed and Muench [28].

### Electron microscopy

A passage 3 virus harvest obtained 4 days after inoculation (12 mL) was concentrated (60-fold) in a Beckmann-Coulter ultracentrifuge at 30,000 x *g* for 16 hours at 4°C, suspended in 200μL L15 medium (Life Technologies), and used for negative staining and cryo transmission electron microscopy (EM). For negative staining EM, 10 μl of a 1:2 mixture of virus suspension and 3% (w/v) ammonium molybdate, pH 6.8, was applied to a 100 mesh copper grid with carbon film. After 1 minute incubation excess was removed by blotting. Specimen were observed and photographed in a JEOL JEM2100 transmission electron microscope (JEOL Ltd, Japan) equipped with a Gatan US4000 camera. For electron tomography of negative stained virus particles, 10 nm gold particles were added as fiducials to the virus mixture to facilitate alignment. A series of (two times binned) images were recorded from virus particles at magnifications of 30.000 to 50.000 times by tilting the specimen from -65 to +65 with increments of 1. The images were processed using a fiducial-bead based alignment procedure and back-projection algorithm, as implemented in the IMOD reconstruction package, to convert the information in the series of tilted projection images into a 3D tomogram [29]. For cryo-EM, 4 μl of the virus suspension was applied to a holey carbon grid. The specimen was then blotted and vitrified in liquid ethane using a Vitrobot (FEI Company). Frozen specimen were observed at -180°C using a Gatan CT3500 cryoholder. Images were taken at 2–4 μm underfocus.

### Chloroform treatment

Chloroform treatment of cultured virus harvest was applied to destroy a potential lipid membrane. A virus harvest of 7.5 ^10^log TCID_50_/mL was mixed and incubated with 0% (vol/vol), 10% (vol/vol) and 50% (vol/vol) chloroform for one hour at 4°C, and subsequently spun at 1000 x *g* for 10 minutes at 4°C. The water phase, and the 10^−1^ to 10^−3^ dilutions thereof, were titrated on SK21 cells as described above, and TCID_50_/mL was determined.

### Expression of the major capsid protein in *Escherichia coli*

An 83 kDa fusion protein construct with an N-terminal Glutathione-S-transferase and an internal histidine-tag for immobilized metal affinity chromatography purification was generated in *E*. *coli*. The major capsid protein (MCP) encoding sequence was synthesized (Genscript) and obtained in a plasmid backbone. An EcoRI-HindIII fragment was cloned in pET41A+ (Novagen). The resulting plasmid (pET41a+ GST-his-SDDV-MCP) was transformed to *E*.*coli* Bl21star (DE3) using standard transformation protocols and subsequently cultured on plates and in liquid culture at 37°C in Animal Component-free Luria Bertani medium (LBACF) containing antibiotics. A 500 mL baffled plastic Erlenmeyer flask with 100 mL Terrific Broth-medium Kan50 was inoculated with 1mL of the freshly grown overnight culture of transformed *E*.*coli*. The culture was incubated at 37°C and 175 rpm until it reached O.D. 600 nm ≈ 0.500. At this point, IPTG was added to a final concentration of 1 mM and the culture was incubated for 2.5 hours. The culture was centrifuged at 5,000 rpm for 5 minutes at 4°C, and the pellet was stored at –20°C. The bacterial pellet was thawed on ice and resuspended in 2 mL PBS. Lysozyme (100μg), benzonase (1000U) and MgCl_2_ (until 10mM) were added to improve lysis and reduce viscosity. The bacteria were sonicated on ice until a homogeneous suspension was obtained, mixed gently and incubated at RT for 1.5 hours. Thirteen mL denaturing lysis buffer (50 mM TRIS-HCl, 300 mM KCl, 6 M Urea pH 8.0) was added. The lysate was incubated at 4°C for 1 hour, sonicated again, and centrifuged at 9000 x *g* for 10 minutes at 4°C. The supernatant was transferred to a 15 mL tube and centrifuged for 30 minutes at 9000 x *g* and 4°C. After passage through a 0.45 μm filter, protein was purified from the supernatant using IMAC cartridges (BioRAD laboratories cat. no. 732–4612). The default denaturing IMAC procedure was carried out on a BioRAD Profinia apparatus. The GST-his-SDDV-MCP molecules that bound to the IMAC cartridge were eluted from the column using 15 mL of denaturing elution buffer (50 mM TRIS-HCl, 300mM KCl, 6 M Urea, 250 mM imidazole, pH 8.0). Urea and imidazole were removed from the elution fraction by dialysis against 1 liter of PBS pH 7.4. The concentration of the dialyzed GST-his-SDDV-MCP solution was determined by comparison with a BSA-dilution series on an SDS-page gel (S5 Fig). The density of the signals was measured by GeneTools software from Syngene.

### Infection of *L*. *calcarifer*

SK21 cells were seeded at 3.0 x 10^4^/cm^2^ and incubated for 24 hours at 28°C. The overnight culture medium was removed from the flask. Subconfluent monolayers at a density of 3.5–4.0 x 10^4^ cells/cm^2^ were infected with an MOI of 0.01 TCID_50_ per cell in a reduced volume (0.5 mL). The cells were incubated at 28°C and ambient CO_2_ for 30 min. The inoculum was removed, fresh culture medium was added and cells were cultured until >50% CPE was observed at day 3 after inoculation. The virus was harvested by one freeze-thaw cycle at -70°C and 4°C, and subsequently cleared from cell debris by centrifugation at 1000 x *g* for 5 minutes at 4°C. The harvest was stored at -70°C until infection.

The undiluted harvest was used for infection of fish (0.1 mL/fish of undiluted SDDV: 5.5 x 10^6^ TCID_50_ per fish for intraperitoneal (IP) injection; 0.01 mL/fish of undiluted SDDV: 5.5 x 10^5^ TCID_50_ per fish for intramuscular (IM) injection). A 1:10 dilution of culture harvest in dilution buffer (PBS + 1.5% NaCl) was used for IP infection of 5.5 x 10^5^ TCID_50_ per fish to match the IM infection (group 1–4). Control fish (group 5) were injected with dilution buffer only. Fish were infected at an average weight of 21 g.

A total of 460 fish were available: four groups of 95 fish were kept in four separate tanks for infection, and 80 fish in a fifth tank served as controls. Tanks were filled with sea water (30 ppt) of 28°C ± 2°C. Fish were starved for at least 36 hours prior to IP or IM infection to empty the gastro-intestinal tract in order to reduce the risk of damage to internal organs during injection. Immediately before the infection, 20 fish from each group were weighed together to obtain the average body weight for each group. All fish were anaesthetized using AQUI-S (AQUI-S, New Zealand) prior to the inoculation procedure. Fish were fed ad libitum from the day after infection.

Infected fish (groups 1–4) were kept in four separate 250L tanks. A vertical net was installed in each 250L tank to create partitions of 1/3 and 2/3 of the tank. The 1/3 partition held 15 fish for mortality observation. The other 2/3 (80 fish) were used for harvesting of sera and organs over the time course of the experiment. Uninfected control fish (group 5) were housed in one 250L partition of a separate 500L tank to align water temperature with the infected fish. From each of the 5 groups, sera of 15 fish were sampled at time point day 1 (dissemination control), 3, 7, 10 and 14 post-infection (total 75 fish). Excess fish (5 for each group, if without mortalities) were culled at day 14 for collection of serum. The 15 fish in the observation tank were kept until day 21 to evaluate mortality of each infection method. At each sampling time point, the serum was pooled by group.

### Virus reisolation from experimentally infected *L*. *calcarifer*

Pooled sera of experimentally infected fish (see above), harvested at day 7 and day 10 after infection, were screened for presence of infectious virus. Sera were diluted 1:100 (v/v) and 1:1000 (v/v) in culture medium for inoculation of SK21 cells, which was carried out as described above. If no CPE occurred in the first passage of the virus, a second passage was performed.

### Vaccine production and inactivation procedures

Virus for vaccine production was cultured as described above. Cultures were harvested with one freeze-thaw cycle and stored at -70°C until inactivation. Formalin inactivation was performed by diluting formalin (37% (w/w) formaldehyde) 10 times to 3.7% (w/w) by mixing with water. This diluted stock was diluted another 100 times with cool (4°C) virus culture harvest to a final concentration of 0.037% (w/w) formaldehyde. The mixture was continuously stirred during an inactivation period of 10 days at 4°C. Binary ethyleneimine (BEI) inactivation was performed by activating 1.09 M bromoethylamine hydrobromide (BEA) with 1.91 M NaOH 1:1 (v/v) and subsequent incubation of 1 hour at 37°C to achieve a BEI concentration of 0.55 M. The culture harvest was inactivated at a final concentration of 0.01 M BEI. The pH of the mixtures was checked 2 and 21 hours after BEI addition and adjusted to be within the range of 7.4–7.65 by adding 1.0 M NaOH or 1.0 M HCl. The total Incubation time was 45 hours at 37°C. BEI was neutralized by adding sodiumthiosulphate to the mixture, and the pH was adjusted to be within the range of 7.4–7.65 as described above. The formalin- and BEI-inactivated culture harvests were titrated on SK21 cells to confirm successful inactivation of the virus. Three serial passages of the inactivated material confirmed absence of live virus in the inactivated harvest. The inactivated virus preparations were stored at 4°C until vaccine formulation.

### Vaccine formulation

Vaccines were formulated in MONTANIDE ISA 763A VG oil (Seppic), a water-in-oil emulsion based on non-mineral oils. The preparation of the emulsion was performed by mixing the water phase into the oil phase at 1000 rpm (mixing velocity). The water phase consisted of inactivated virus harvest in culture medium, or purified recombinant protein in PBS. In Table 1 an overview of vaccines and antigen concentrations after formulation is provided.

### Vaccination—challenge

Five groups of 25 Asian seabass (60 g) were randomly assigned to treatment groups (vaccines see Table 1). Fish were vaccinated with 0.1 mL of the prototype vaccines by intraperitoneal injection on day 0. Negative control fish were injected with 0.1 mL of placebo vaccine (vaccine dilution buffer PBS + 1.5% NaCl, formulated in ISA 763A VG oil as described above). Fish were starved for at least 36 hours prior to the vaccination. Immediately before the vaccination, fish from each group were weighed together to obtain the average body weight for each group. All fish were anaesthetized using AQUI-S (AQUI-S, New Zealand) prior to the vaccination procedure. Fish were fed ad libitum from the day after vaccination. Vaccinated fish were kept in 250 L partitions of 500 L tanks that were created by installing a vertical net in the tank. On day 28 post vaccination, all fish were challenged. The challenge dose was 2.0 x 10^7^ TCID_50_/fish and the fish were subsequently kept in 125 L partitions of 250 L tanks that were created by installing a vertical net in the tank. Mortalities were recorded daily up to 28 days post challenge.

### Survival curves

Kaplan Meier survival curves were constructed using SPSS v22 (IBM). Analysis of the similarity of the curves was performed using the Tarone-Ware test.

### Calculation of relative percent survival

The relative protection percentage (RPS) in the vaccination-challenge study was calculated as follows. The ‘percentage protected control’ was calculated as the number of survivors in the control group, divided by the total number of control fish, multiplied by 100% (% protected control = [# survivors control] / [total # control fish] * 100% = x %). The ‘percentage protected in the vaccine group’ was calculated as the number of survivors in the vaccine group, divided by the total number of vaccinated fish, multiplied by 100% (% protected vaccine group = [# survivors vaccine] / [total # vaccinated fish] * 100% = y %). The RPS is formulated as *RPS = 1-*(*% protection test / % protected control*) or in a mathematical formulation RPS = 1-(x/y)* 100%.

### Virus deposit

A representative virus has been deposited with the Collection Nationale de Cultures de Microorganisms (CNCM), Institut Pasteur, 25 Rue du Docteur Roux, F-75724 Paris Cedex 15, France, under accession number CNCM I-4754)

## Supporting Information

S1 Fig Dot plot analysis of SDDV with representative members of the *Iridoviridae*. (PDF)

S2 Fig Phylogenetic clustering of SDDV within the *Iridoviridae* family. The deduced amino acid sequences of the major capsid protein encoding gene was aligned with the corresponding gene of all viruses of which this gene has been sequenced. The neighbor-joining method with pairwise deletion within the MEGA-5 package was used, bootstrap values (for 500 replications) are provided at the root of the clusters and the scale bar is a measure of the proportion of divergence. A • indicates SDDV.
(PDF)

S3 Fig CPE on Seabass Brain cells. Confluent monolayer at day 10 post inoculation of controls cells (A) and SDDV infected cells (B). Note round-up cells, which can clearly be distinguished from normal, attached cells on the monolayer. The culture was harvested and the ^10^log TCID_50_ was 6.5.
(PDF)

S4 Fig Sensitivity of SDDV to chloroform treatment. Virus (7.5 ^10^Log TCID_50_/mL) was incubated with 10% or 50% chloroform. A 100-fold decrease in infectivity was observed due to incubation with chloroform. The remaining infectivity of 5,5 ^10^Log TCID_50_ /mL indicates that a lipid envelope is not essential for replication of the SDDV.
(PDF)

S5 Fig Analysis and quantification of GST-his-SDDV MCP after purification. Lane 1, Protein size marker, Lane 2 to 4: concentration range of BSA used for quantification 25-50-100 μg/lane; Lane 5: SDDV-MCP undiluted; Lane 6: SDDV-MCP 2x diluted.
(PDF)

S1 Table SDDV qPCR on serum and tissue samples of fish with scale drop syndrome and healthy control fish. N/A: Below the threshold, so no virus detected; POS: positive; NEG: Negative.
(PDF)

S2 Table Genome location of conserved genes within the *Iridoviridae* family. ^a^SDDV: scale drop disease virus; ISKNV: infectious spleen and kidney necrosis virus; OSGIV: orange-spotted grouper iridovirus; RBIV: rock bream iridovirus; TRBIV: turbot reddish body virus; RSIV: red sea bream iridovirus; LCDV-1: lymphocystis disease virus 1; LCDV-C: lymphocystis disease virus China; SGIV: Singapore grouper iridovirus; GIV: grouper iridovirus; FV3: Frog virus 3; STIV: soft-shelled turtle iridovirus; TFV: tiger frog virus; ATV: Ambystoma tigrinum virus; EHNV: epizootic haematopoietic necrosis virus; IIV-6: invertebrate iridovirus type 6;IIV-3: invertebrate iridescent virus 3. ^b^Gene name according to Eaton et al. (reference [9] in main text).
(PDF)

S3 Table CPE positive SK21 cultures contain SDDV. (PDF)

S4 Table Re-isolation of SDDV from serum of experimentally infected fish (4th Koch’s postulate). (PDF)

S5 Table Presence/absence of SDDV-DNA in serum of vaccinated fish on day 28. (PDF)

S6 Table Primer and probe sequences used for SDDV qPCR and RSIV PCR. (PDF)
