# Supplementary material for: A Novel Virus Causes Scale Drop Disease in Lates calcarifer
Source: PLoS Pathog. 2015 Aug 7;11(8):e1005074. doi: 10.1371/journal.ppat.1005074 (PMC4529248; doi:10.1371/journal.ppat.1005074)

S1 Figure

Dotplot SDDV – *Megalocytivirus*

RBIV (AY532606)-SDDV

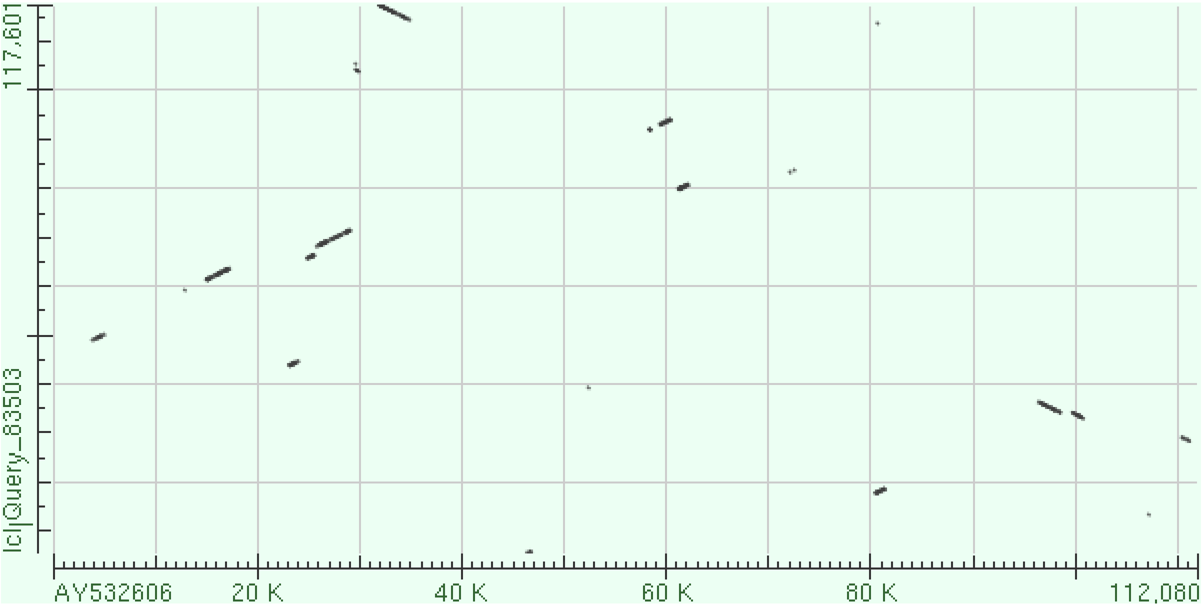

OSGIV (AY894343.1)-SDDV

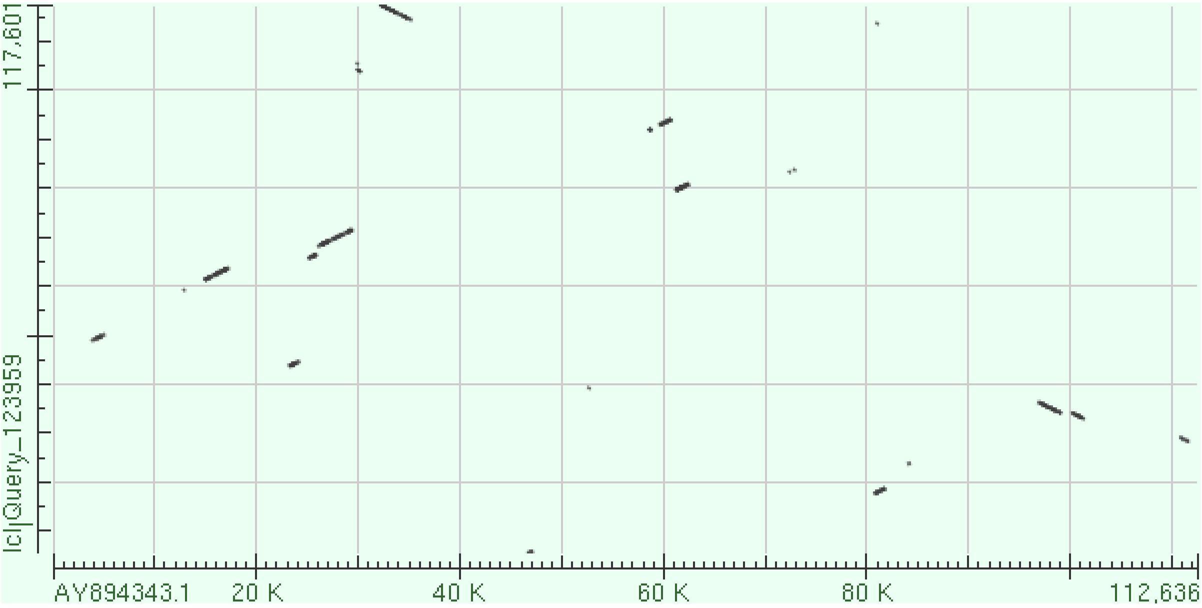

RSIV (BD143114)-SDDV

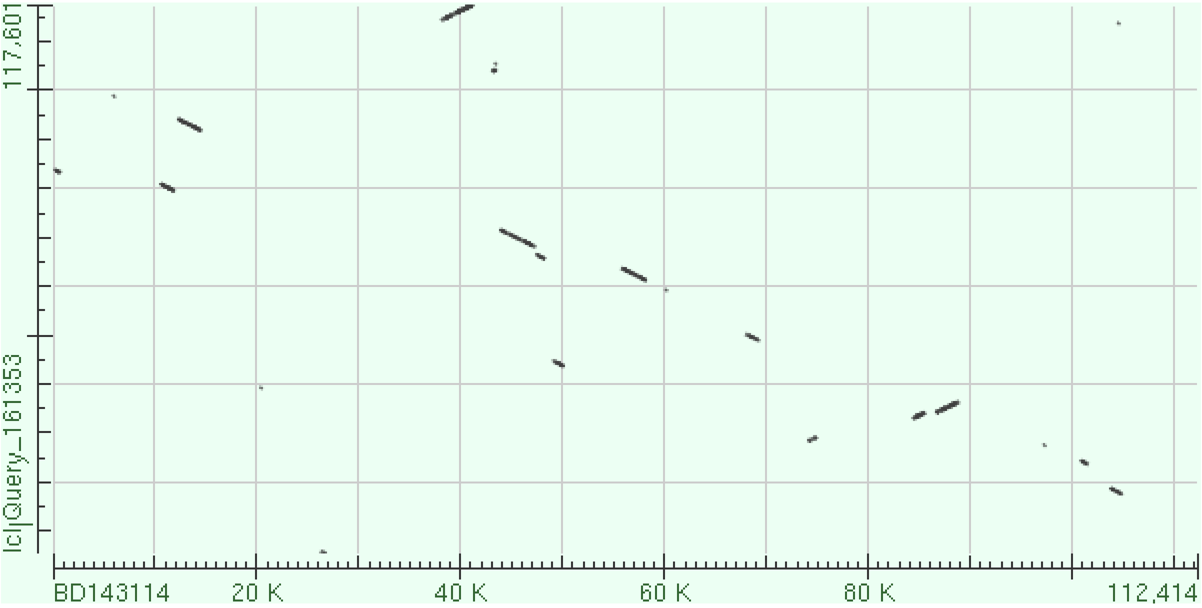

Dotplot SDDV – *Megalocytivirus* (continued)

ISKNV (NC_003494.1)-SDDV

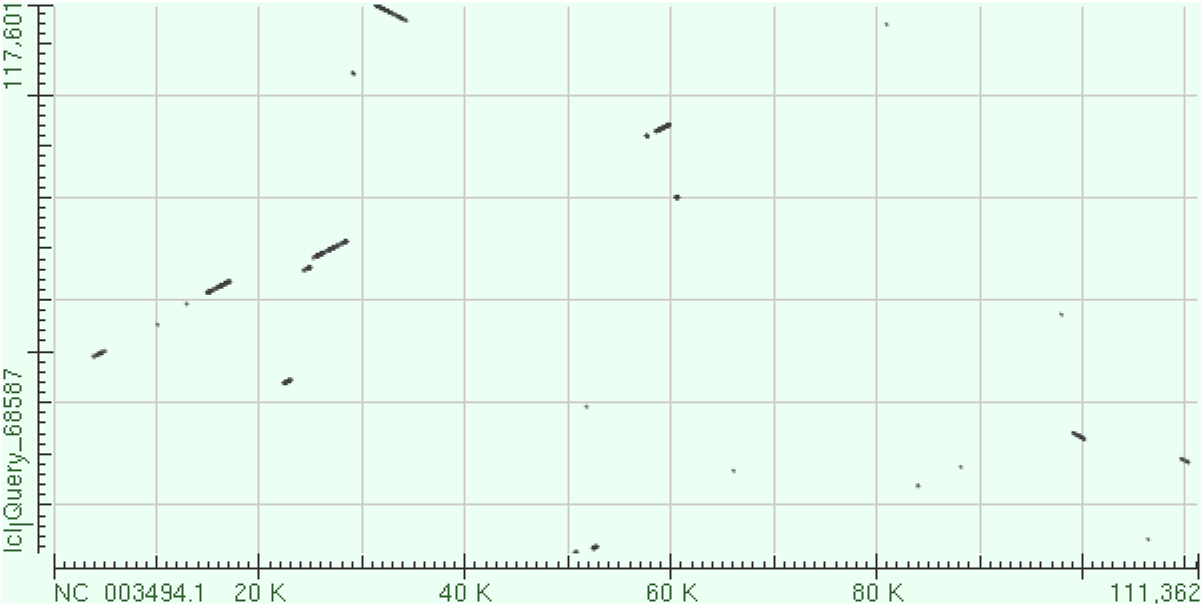

TRBIV (GQ273492.1)-SDDV

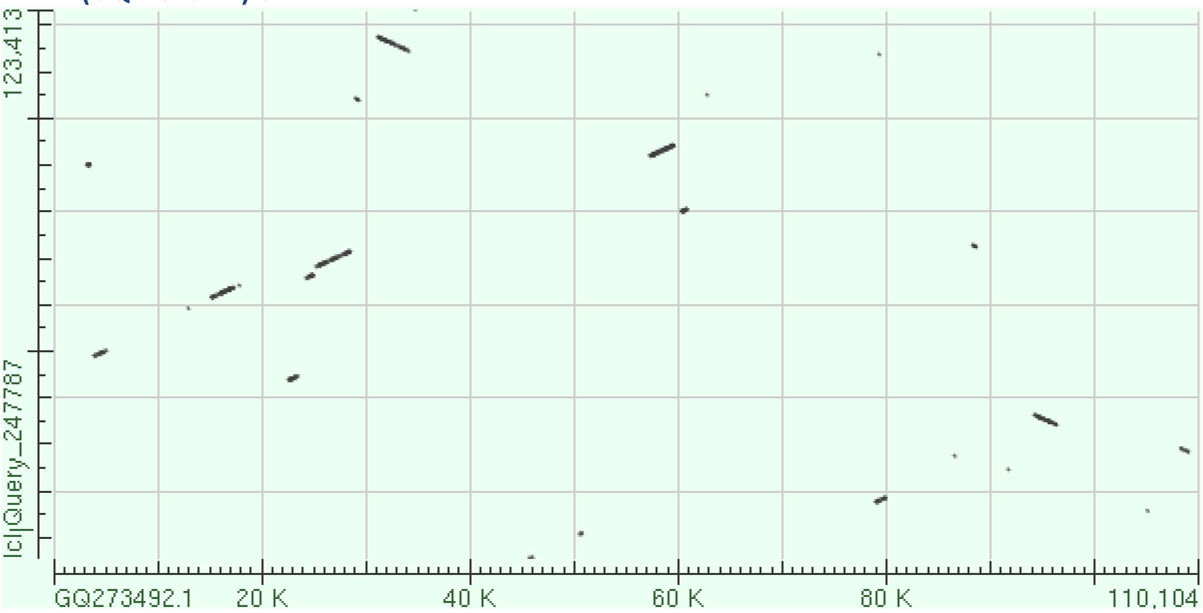

Dotplot SDDV – *Chloriridovirus*

IIV-3 (NC_008187.1)-SDDV

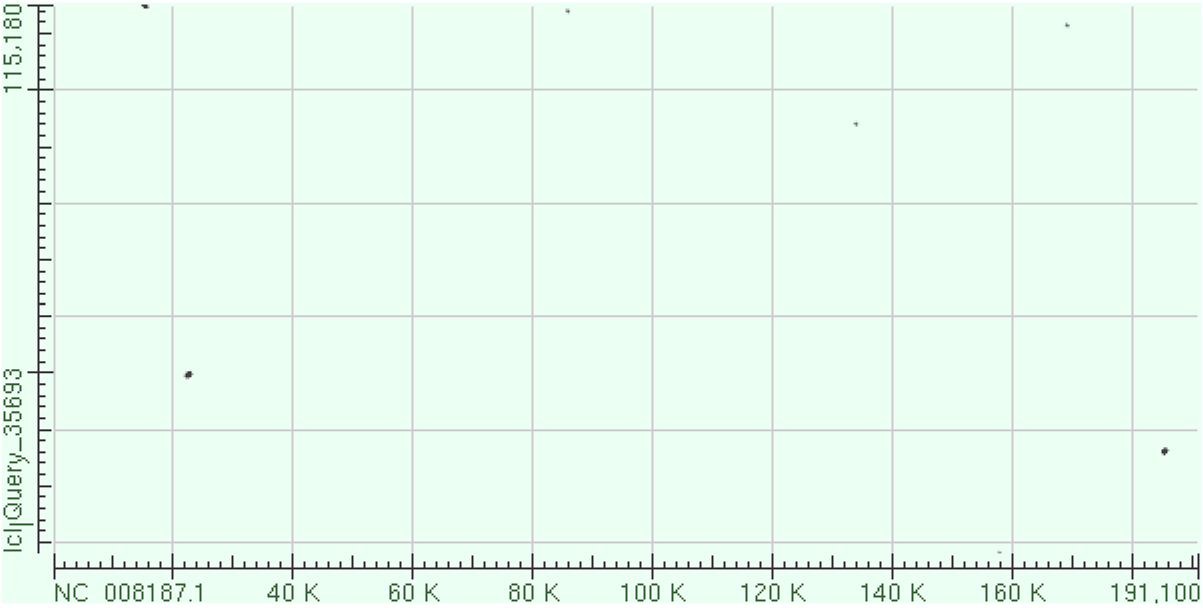

Dotplot SDDV – *Iridovirus*

IIV-6 (NC_003038.1)-SDDV

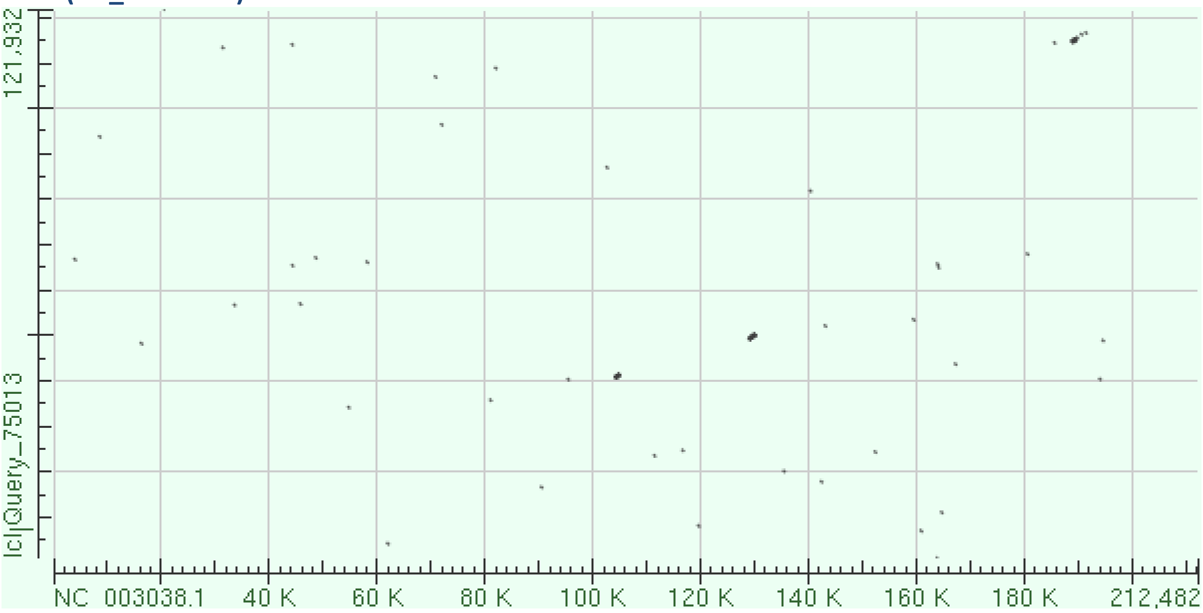

Dotplot SDDV – *Ranavirus*

ATV (NC_005832.1)-SDDV

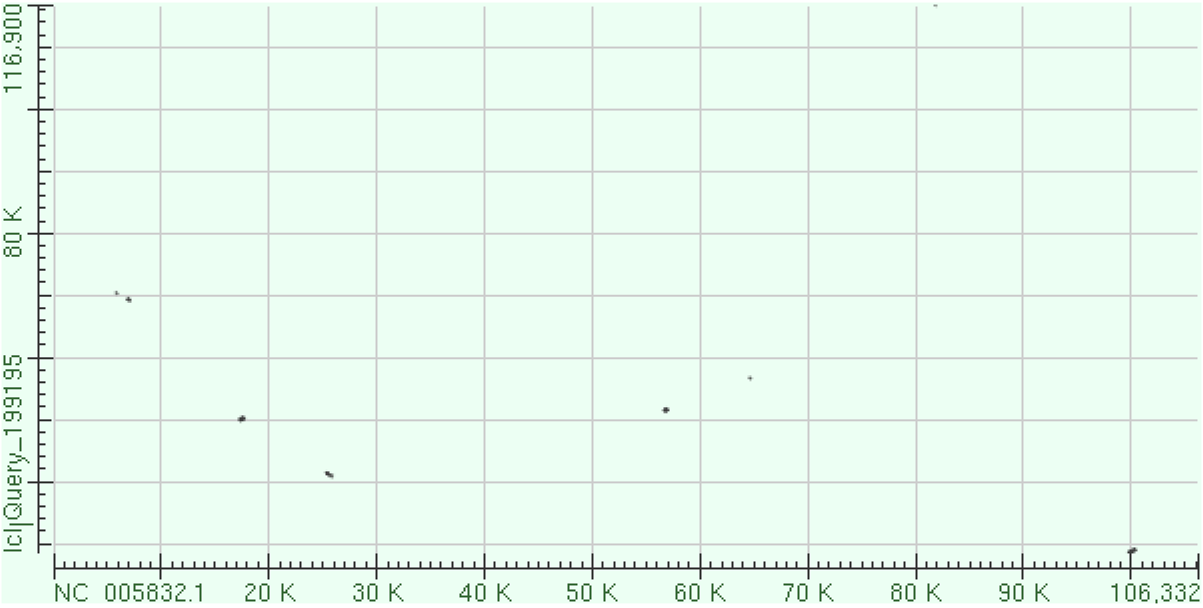

SGIV (NC_006549.1)-SDDV

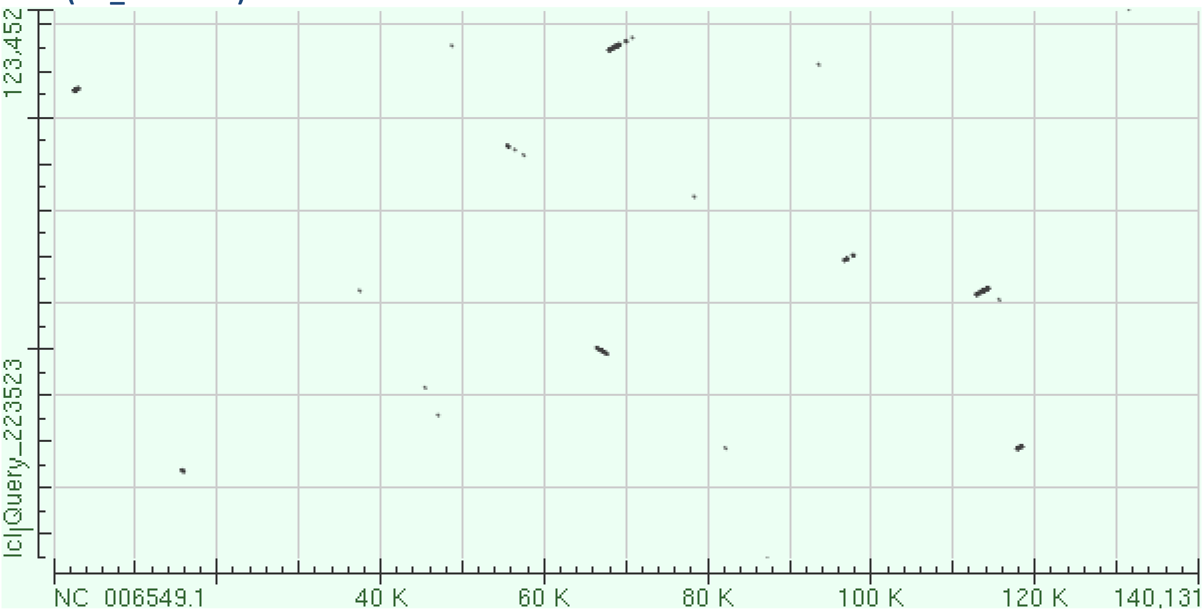

Dotplot SDDV – *Lymphocystivirus*

LCDV china (NC_005902)-SDDV

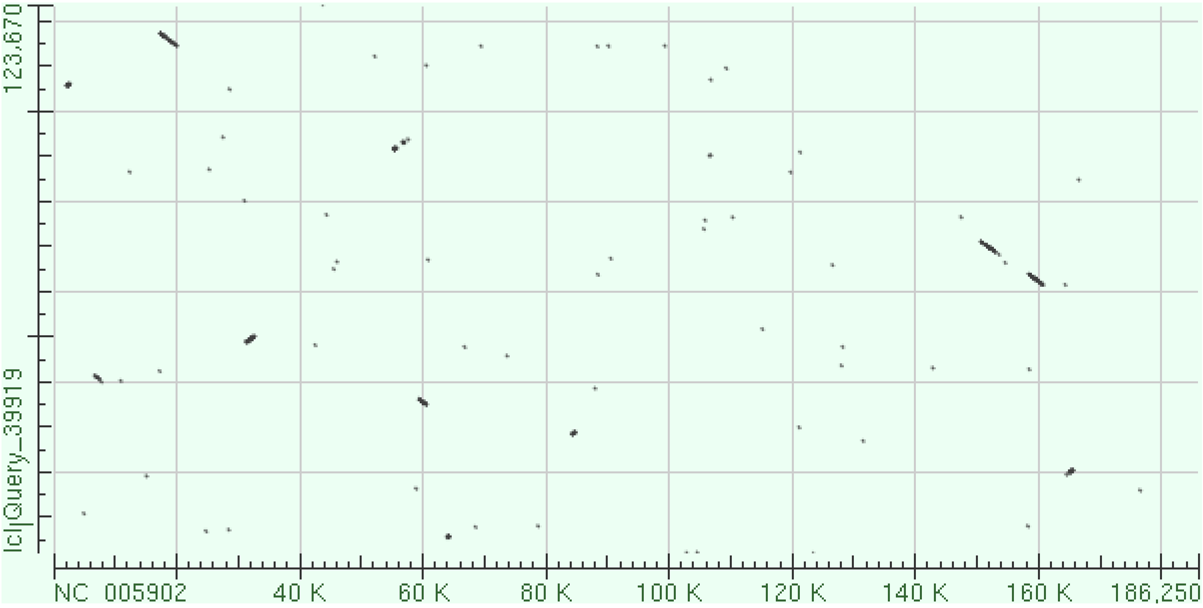

LCDV-1 (NC_001824.1)-SDDV

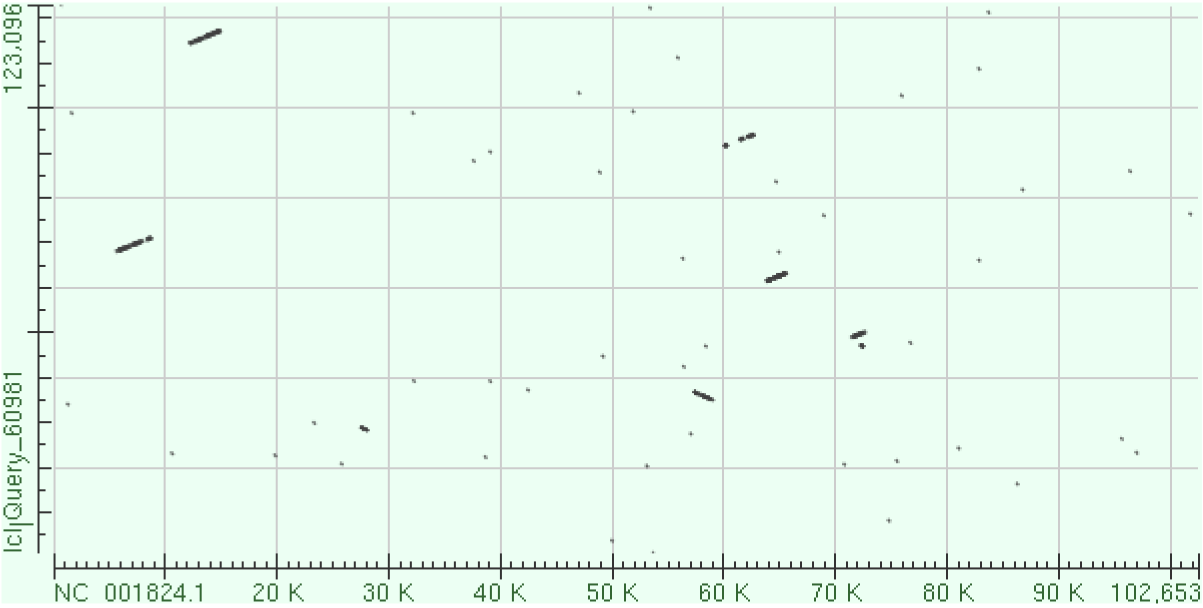

Dotplot SDDV – SDDV

SDDV-SDDV

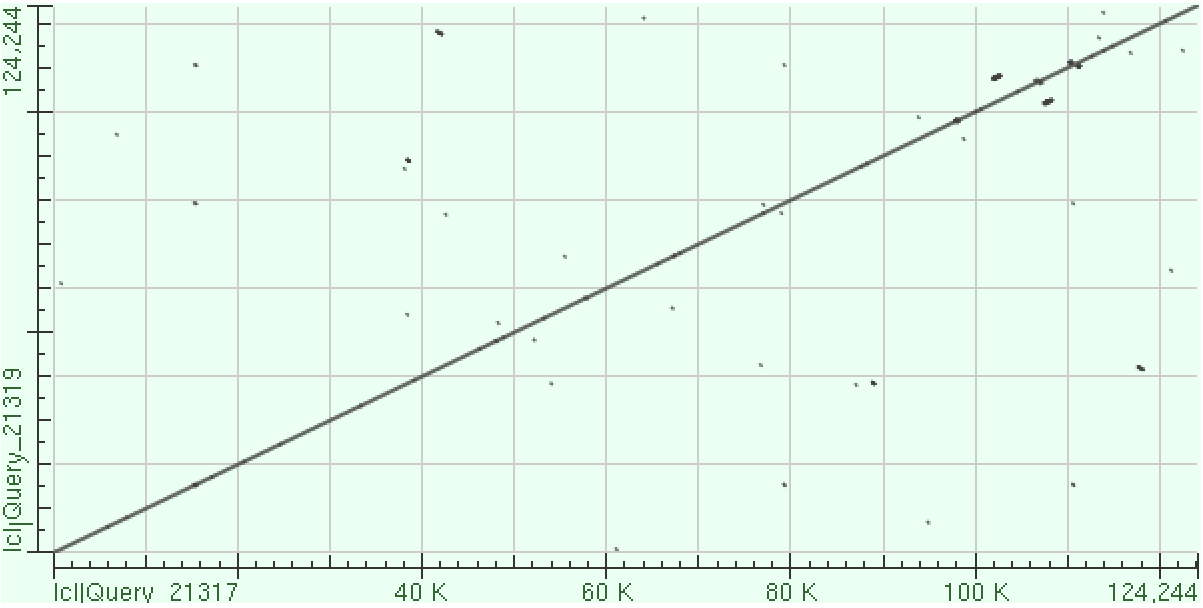

Supplement: S1 Fig — (PDF) [file ppat.1005074.s001.pdf]
