# Supplementary material for: A Novel Virus Causes Scale Drop Disease in Lates calcarifer
Source: PLoS Pathog. 2015 Aug 7;11(8):e1005074. doi: 10.1371/journal.ppat.1005074 (PMC4529248; doi:10.1371/journal.ppat.1005074)
Supplement: S2 Fig — The deduced amino acid sequences of the major capsid protein encoding gene was aligned with the corresponding gene of all viruses of which this gene has been sequenced. The neighbor-joining method with pairwise deletion within the MEGA-5 package was used, bootstrap values (for 500 replications) are provided at the root of the clusters and the scale bar is a measure of the proportion of divergence. A • indicates SDDV. (PDF) [file ppat.1005074.s002.pdf]

S2 Figure

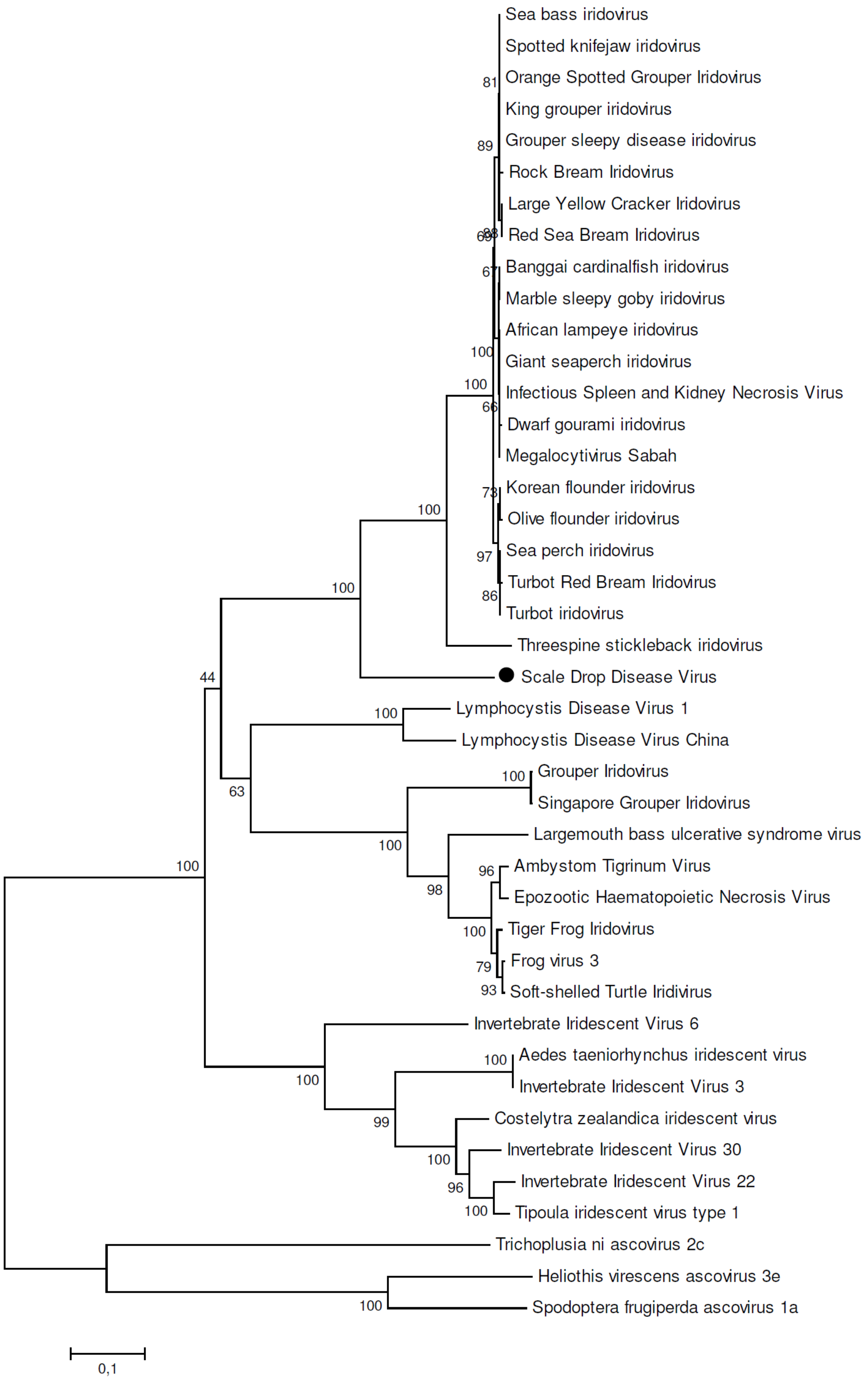

Figure S2: The deduced amino acid sequences of the major capsid protein encoding gene was aligned with the corresponding gene of all iridoviruses of which this gene has been sequenced. The neighbor-joining method with pairwise deletion within the MEGA-5 package was used, bootstrap values (for 500 replications) are provided at the root of the clusters and the scale bar is a measure of the proportion of divergence. A • indicates SDDV.
