# Supplementary material for: A Novel Virus Causes Scale Drop Disease in Lates calcarifer
Source: PLoS Pathog. 2015 Aug 7;11(8):e1005074. doi: 10.1371/journal.ppat.1005074 (PMC4529248; doi:10.1371/journal.ppat.1005074)
Supplement: S3 Fig — Confluent monolayer at day 10 post inoculation of controls cells (A) and SDDV infected cells (B). Note round-up cells, which can clearly be distinguished from normal, attached cells on the monolayer. The culture was harvested and the 10log TCID50 was 6.5. (PDF) [file ppat.1005074.s003.pdf]

S3 Fig CPE on Seabass Brain cells.

A

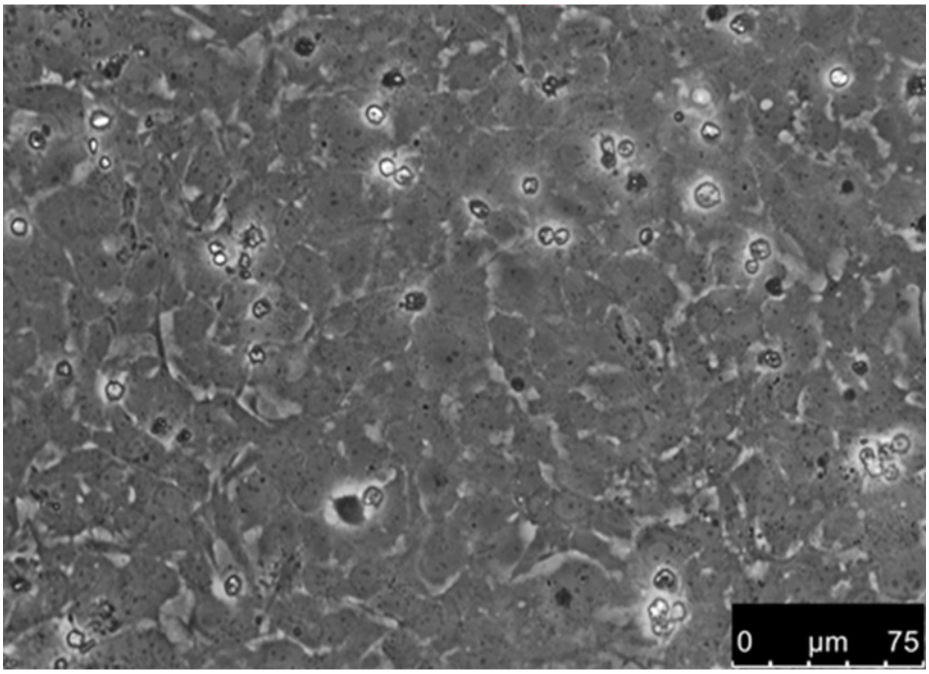

B

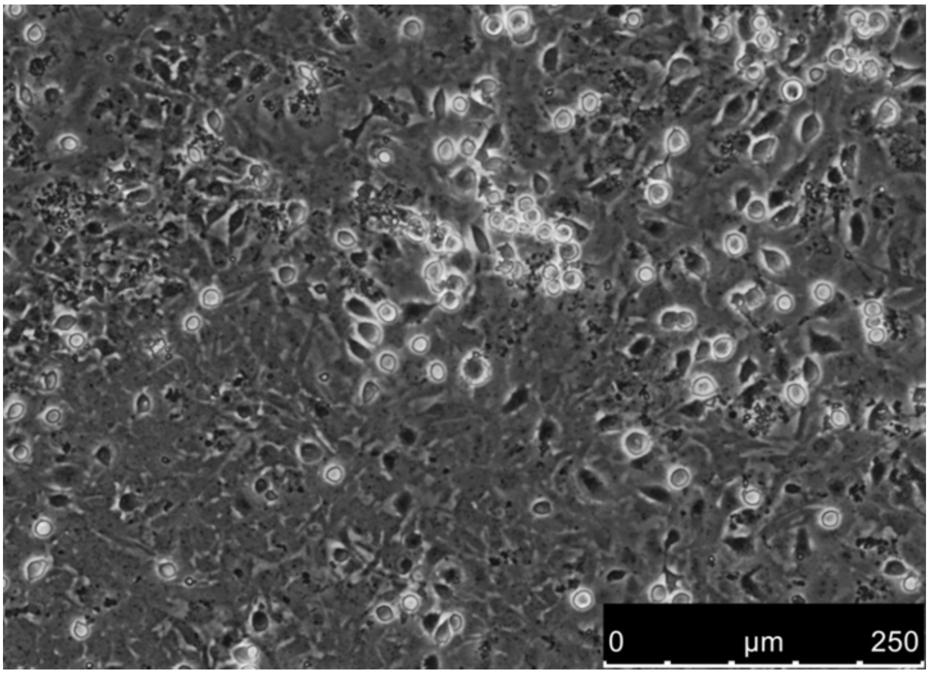

S3 Fig legend: CPE on Seabass Brain cells. Confluent monolayer at day 10 post inoculation of controls cells (A) and SDDV infected cells (B). Note round-up cells, which can clearly be distinguished from normal, attached cells on the monolayer. The culture was harvested and the ^{10}log $TCID_{50}$ was 6.5.
