# Supplementary material for: A Novel Virus Causes Scale Drop Disease in Lates calcarifer
Source: PLoS Pathog. 2015 Aug 7;11(8):e1005074. doi: 10.1371/journal.ppat.1005074 (PMC4529248; doi:10.1371/journal.ppat.1005074)
Supplement: S4 Fig — Virus (7.5 10Log TCID50/mL) was incubated with 10% or 50% chloroform. A 100-fold decrease in infectivity was observed due to incubation with chloroform. The remaining infectivity of 5,5 10Log TCID50 /mL indicates that a lipid envelope is not essential for replication of the SDDV. (PDF) [file ppat.1005074.s004.pdf]

S4 Fig. Sensitivity of SDDV to chloroform treatment.

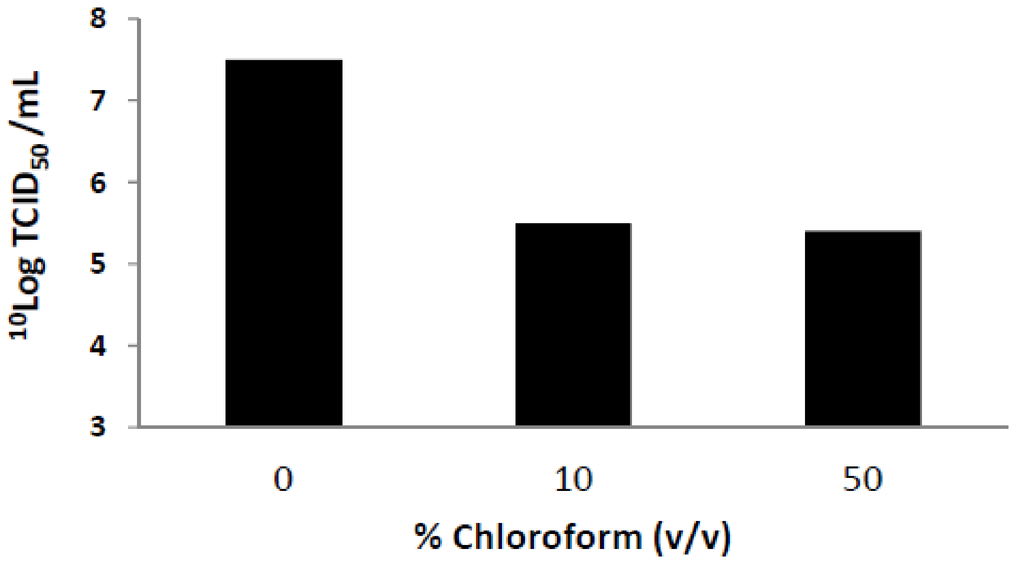

Sample treatment	^{10}Log TCID$_{50}$ per mL	Mean ^{10}Log TCID$_{50}$ per mL
Chloroform 0%	7.42-7.50*	7.46
Chloroform 10%	5.49-5.57	5.53
Chloroform 50%	5.29-5.41	5.35

* Results of duplicate experiments

Legend figure S4. Sensitivity of SDDV to cloroform treatment. Virus (7.5 ^{10}Log TCID$_{50}$/mL) was incubated with 10% or 50% chloroform. A 100-fold decrease in infectivity was observed due to incubation with chloroform. The remaining infectivity of 5,5 ^{10}Log TCID$_{50}$ /mL indicates that a lipid envelope is not essential for replication of SDDV.
