# Supplementary material for: A Novel Virus Causes Scale Drop Disease in Lates calcarifer
Source: PLoS Pathog. 2015 Aug 7;11(8):e1005074. doi: 10.1371/journal.ppat.1005074 (PMC4529248; doi:10.1371/journal.ppat.1005074)
Supplement: S5 Fig — Lane 1, Protein size marker, Lane 2 to 4: concentration range of BSA used for quantification 25-50-100 μg/lane; Lane 5: SDDV-MCP undiluted; Lane 6: SDDV-MCP 2x diluted. (PDF) [file ppat.1005074.s005.pdf]

S5 Figure. Analysis and quantification of GST-his-SDVV-MCP (rec-MCP) after purification

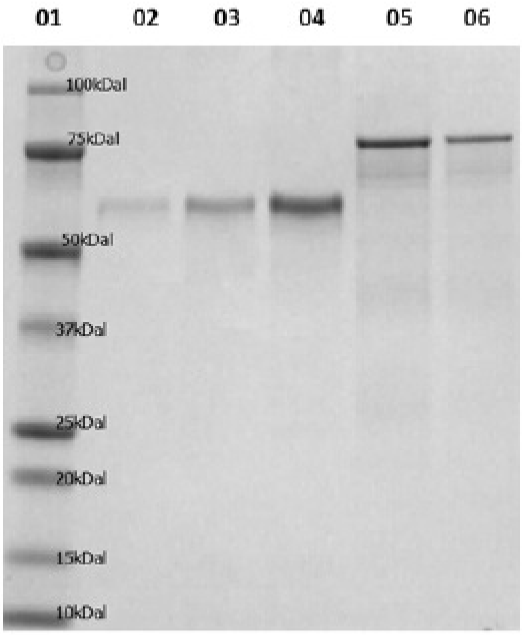

Lane 1, Protein size marker, Lane 2 to 4: concentration range of BSA used for quantification 25-50-100 µg/lane; Lane 5: SDDV-MCP undiluted; Lane 6: SDDV-MCP 2x diluted.
