# Supplementary material for: A Novel Virus Causes Scale Drop Disease in Lates calcarifer
Source: PLoS Pathog. 2015 Aug 7;11(8):e1005074. doi: 10.1371/journal.ppat.1005074 (PMC4529248; doi:10.1371/journal.ppat.1005074)
Supplement: S1 Table — N/A: Below the threshold, so no virus detected; POS: positive; NEG: Negative. (PDF) [file ppat.1005074.s006.pdf]

S1 Table: SDDV qPCR on serum and tissue samples of fish with scale drop syndrome and healthy control fish

	Treshold cycle (Cq)	Quantity (copies/µl)	qPCR
**Singapore, December 2010**			
Scale drop syndrome serum 1	23.5	4.4E4	POS
Scale drop syndrome serum 2	22.3	1.0E5	POS
Scale drop syndrome spleen 2	21.9	1.3E5	POS
Scale drop syndrome kidney 2	23.9	3.7E4	POS
Scale drop syndrome kidney 3	21.5	2.0E5	POS
**Singapore, July 2011**			
Scale drop syndrome serum 3	N/A	N/A	NEG
Scale drop syndrome serum 4	26.4	5.0E3	POS
Healthy fish serum 1	N/A	N/A	NEG
Healthy fish serum 2	N/A	N/A	NEG
Scale drop syndrome heart	23.2	6.0E4	POS
Scale drop syndrome spleen 1	25.0	1.4E4	POS
Scale drop syndrome kidney 1	22.9	7.0E4	POS
Healthy fish kidney 1	N/A	N/A	NEG
Healthy fish kidney 2	N/A	N/A	NEG
Healthy fish kidney 3	N/A	N/A	NEG
Healthy fish kidney 4	N/A	N/A	NEG
Healthy fish spleen 1	N/A	N/A	NEG
Healthy fish spleen 2	N/A	N/A	NEG
**Indonesian serum samples, June 2012**			
Healthy fish 1	N/A	N/A	NEG
Healthy fish 2	N/A	N/A	NEG
Healthy fish 3	N/A	N/A	NEG
Early scale drop syndrome 1	25.5	1.5E4	POS
Early scale drop syndrome 2	22.8	5.1E4	POS
Early scale drop syndrome 3	22.4	7.6E4	POS
Early scale drop syndrome 4	21.7	1.2E5	POS
Early scale drop syndrome 5	N/A	N/A	NEG
Late scale drop syndrome 1	23.0	9.1E4	POS
Late scale drop syndrome 2	29.2	6.5E2	POS
Late scale drop syndrome 3	24.1	2.0E4	POS
Late scale drop syndrome 4	24.7	1.3E4	POS
Late scale drop syndrome 5	23.0	4.8E4	POS
**Indonesian serum samples, November 2012**			
Scale drop syndrome fish 1	N/A	N/A	NEG
Scale drop syndrome fish 2	N/A	N/A	NEG
Scale drop syndrome fish 3	N/A	N/A	NEG
Scale drop syndrome fish 4	22.0	1.7E5	POS
Scale drop syndrome fish 5	24.3	3.3E4	POS
Scale drop syndrome fish 6	33.9	3.9E1	NEG
Scale drop syndrome fish 7	22.4	1.3E5	POS
Scale drop syndrome fish 8	29.4	9.1E2	POS
Scale drop syndrome fish 9	30.9	3.1E2	POS
Scale drop syndrome fish 10	29.4	8.8E2	POS
Scale drop syndrome fish 11	20.4	5.0E5	POS
Scale drop syndrome fish 12	21.7	2.1E5	POS
Scale drop syndrome fish 13	21.0	3.4E5	POS
Scale drop syndrome fish 14	18.6	1.8E6	POS
Scale drop syndrome fish 15	19.7	8.6E5	POS
Scale drop syndrome fish 16	20.1	6.2E5	POS
Scale drop syndrome fish 17	20.3	5.5E5	POS
Scale drop syndrome fish 18	21.6	2.1E5	POS
Scale drop syndrome fish 19	20.0	6.7E5	POS
Scale drop syndrome fish 20	21.5	2.4E5	POS
Healthy fish 1	N/A	N/A	NEG
Healthy fish 2	N/A	N/A	NEG
Healthy fish 3	N/A	N/A	NEG

N/A: below the threshold, therefore no detectable virus; POS: positive; NEG: Negative
