# Supplementary material for: A Novel Virus Causes Scale Drop Disease in Lates calcarifer
Source: PLoS Pathog. 2015 Aug 7;11(8):e1005074. doi: 10.1371/journal.ppat.1005074 (PMC4529248; doi:10.1371/journal.ppat.1005074)
Supplement: S2 Table — aSDDV: scale drop disease virus; ISKNV: infectious spleen and kidney necrosis virus; OSGIV: orange-spotted grouper iridovirus; RBIV: rock bream iridovirus; TRBIV: turbot reddish body virus; RSIV: red sea bream iridovirus; LCDV-1: lymphocystis disease virus 1; LCDV-C: lymphocystis disease virus China; SGIV: Singapore grouper iridovirus; GIV: grouper iridovirus; FV3: Frog virus 3; STIV: soft-shelled turtle iridovirus; TFV: tiger frog virus; ATV: Ambystoma tigrinum virus; EHNV: epizootic haematopoietic necrosis virus; IIV-6: invertebrate iridovirus type 6;IIV-3: invertebrate iridescent virus 3. bGene name according to Eaton et al. (reference [9] in main text). (PDF) [file ppat.1005074.s007.pdf]

S2 Table . Genome location of conserved genes within the *Iridoviridae* family

			Megalocytivirus					*Lymphocystivirus*		*Ranavirus*							*Iridovirus*	*Chloroiridovirus*
		SDDV[a]	ISKNV	OSGIV	RBIV	TRBIV	RSIV	LCDV-1	LCDV-C	SGIV	GIV	FV3	STIV	TFV	ATV	EHNV	IIV-6	IIV-3
	**Gene name[b]**																	
1	Putative replication factor and/or DNA binding/packing	**17L**	61L	60L	57L	56L	92R	91L	180R	116R	67R	1R	1R	105R	95R	100R	282R	79L
2	Myristilated membrane protein	**30L**	90,5L	88,5L	85L	83R	575R	89L	38R	19R	4R	2L	2L	2L	1L	1L	337L	47R
3	DNA-dep RNA pol-II largest subunit	**76L**	28L	31L	29L	28L	239R	7L	190R	104L	59L	8R	10R	8R	6R	7R	176R\343L	90L
4	Putative NTPase I	**104L**	63L	63L	59L	58L	13R	70L	075L	60R	30R	9L	11L	9L	7L	8L	22L	87L
5	Unkown	**33L**	96L	93L	91.5	88L	550R	57L	100L	118R	68R	12L	14L	12L	91R	95R	287R	56L
6	ATPase-like protein	**35L**	123R	119R	116R	113R	407R	30R	114L	134L	73L	15R	16R	16R	87R	92L	75L	88R
7	Serine-threonine protein kinase	**15L**	55L	56L	53L	52L	111R	5L	45R	39L	17L	19R	21R	19R	84L	89L	380R	10L
8	Helicase family	**16L**	56L	57L	54L	53L	101R	4L	7L	54R	28R	21L	24L	21L	82R	86R	67R	4R
9	D5 family NTPase involved in DNA replication	**40R**	109L	106L	101L	99L	493R	69L	78L	52L	27L	22R	25R	22R	81L	85L	184R	121R
10	Putative tyrosine kinase/lipolysaccharide modifying enzyme	**37R**	114L	111L	109L	105L	463R	110R	172R	78L\81L	44L	27R	31R	29R	60R	62R	179R	35R
11	NIF-NLI interaction factor	**99L**	5L	6L	6L	5L	385R	43L	147L	61R	31R	37R	41R	40R	67R	72R	355R	104L
12	Unkown	**91L**	76L	75L	72L	69L	639R	92R	234R	57L	29L	41R	45R	45R	72R	77R	295L	16R
13	Myristilated membrane protein	**61L**	7L	8L	8L	7L	374R	35L	157R	88L	49L	53R	55R	55R	53L	53L	458R	6R
14	Serine-threonine protein kinase	**65R**	13R	15R	13R	13R	349R	77L	177L	150L	83L	57R	60R	59R	49L	58L	98R	98L
15	DNA pol family B exonuclease	**70R**	19R	22R	20R	20R	317L	72R	202L	128R	71R	61R	63R	63R	46L	44L	37L	120R
16	DNA-dep RNA pol-II second largest subunit	**122L**	34R	36R	33R	33R	224L	13L	25R	73L	40L	63L	64R	65L	44R	43R	428L	9R
17	Ribonucleotide reductase small subunit	**52R**	24R	27R	26R	25R	268L	15L	41L	47L	22L	68L	71L	71L	39R	38R	376L	48L
18	Ribonuclease III	**25R**	87R	85R	83R	80R	596L	74R	186R	84L	46L	81L	87L	85L	25R	24R	142R	101R
19	Transcription elongation factor TFIIS	**126R**	29L	32L	31L	29L	238R	97R	115R	85R	47R	82R	88R	86R	24L	23L	349L	55R
20	Proliferating cell nuclear antigen	**39L**	112R	109R	107R	102R	487L	2L	196L	68L	35L	85R	91R	90R	20L	19L	436R	60L
21	Deoxynucleoside kinase	**125L**	32R	34R	31R	31R	234L	73R	27R	67L	34L	86R	92R	91R	19L	18L	143R	29R
22	Ervl/Alr family	**3L**	43L	45L	44L	42L	156R	55L	141L	70R	37R	89R	94R	94R	16L	16L	347L	96R
23	Major capsid protein	**60L**	6L	7L	7L	6L	380R	80L	43L	72R	39R	91R	96R	96R	14L	14L	274R	14L
24	Immediate early protein ICP-46	**36L**	115R	112R	108R	106R	458L	24L	161R	162L	90L	92R	97R	97R	13L	13L	393L	39R
25	Hypothetical protein - Clostridium tetani	**24R**	86L	84.5R	83.5R	79R	600L	9R	152L	98R	56R	94L	99L	100R	11R	11R	307L	33L
26	Putative XPPG-RAD2 type nuclease	**73L**	27L	30L	28L	27L	256R	107R	168R	97L	55L	96R	100R	101R	10L	10L	369L	76L

- **a: SDDV: scale drop disease virus; ISKNV: infectious spleen and kidney necrosis virus; OSGIV: orange-spotted grouper iridovirus; RBIV: rock bream iridovirus; TRBIV: turbot reddish body virus; RSIV: red sea bream iridovirus; LCDV-1: lymphocystis disease virus 1; LCDV-C: lymphocystis disease virus China; SGIV: Singapore grouper iridovirus; GIV: grouper iridovirus; FV3: Frog virus 3; STIV: soft-shelled turtle iridovirus; TFV: tiger frog virus; ATV: Ambystoma tigrinum virus; EHNV: epizootic haematopoietic necrosis virus; IIV-6: invertebrate iridovirus type 6;IIV-3: invertebrate iridescent virus 3.**
- **b: Gene name accorrding to Eaton et al. (reference [9] in main text).**
