# Supplementary material for: A Novel Virus Causes Scale Drop Disease in Lates calcarifer
Source: PLoS Pathog. 2015 Aug 7;11(8):e1005074. doi: 10.1371/journal.ppat.1005074 (PMC4529248; doi:10.1371/journal.ppat.1005074)
Supplement: S3 Table — (PDF) [file ppat.1005074.s008.pdf]

S3 Table. CPE positive SK21 cultures contain SDDV

Dilution*	CPE	SDDV DNA copies per 2 µl	Dilution	CPE	SDDV DNA copies per 2 µl
10^6	+	5.77E+07	10^7	+	1.37E+07
10^6	+	1.95E+07	10^7	+	1.67E+07
10^6	+	2.12E+07	10^7	+	4.94E+06
10^6	+	1.98E+07	10^7	-	4.54E+02
10^6	+	1.58E+07	10^7	+	3.55E+05
10^6	+	2.11E+07	10^7	+	9.84E+06
10^6	+	2.76E+07	10^7	-	2.19E+05
10^6	+	1.64E+07	10^7	-	4.65E+01
10^6	+	1.71E+07	10^7	-	3.34E+05
10^6	+	3.42E+07	10^7	+	3.61E+06

* Ten SK21 cultures inoculated with 10^{-6}, and 10^{-7} dilutions of a cell culture medium sample from the first passage of SDDV on SK21 cells were analyzed for CPE and SDDV-DNA on day 9 post infection. In all wells with CPE the virus is present in high concentrations, furthermore in two wells without CPE also SDDV DNA was present (10^7 dilution)
