# Supplementary material for: A Novel Virus Causes Scale Drop Disease in Lates calcarifer
Source: PLoS Pathog. 2015 Aug 7;11(8):e1005074. doi: 10.1371/journal.ppat.1005074 (PMC4529248; doi:10.1371/journal.ppat.1005074)
Supplement: S4 Table — (PDF) [file ppat.1005074.s009.pdf]

S4 Table. Re-isolation of SDDV from pooled sera of experimentally infected fish (4th Koch's postulate)

Serum	Serum dilution	
	**1:100**	**1:1000**
	**Time until full CPE on SK21 cells (days)**	
**Intraperitoneal (IP)** day 7 post infection	**3***	**3**
**IP** day 10 post infection	**3**	**3**
**Intramuscular (IM)** day 7 post infection	**3**	**3**
**IM** day 10 post infection	**3**	**3**
**IP + IM** day 7 post infection	**3**	**3**
**IP + IM** day 10 post infection	**3**	**3**
**IP** (1:10) day 7 post infection	**3**	**3**
**IP** (1:10) day 10 post infection	**3**	**10**
**Non-infected** control day 7 post mock infection**	no CPE	no CPE
**Non-infected** control day 10 post mock infection	no CPE	no CPE
SDDV control virus	**3**	**3**
SDDV control virus	**3**	**3**

* Presence of SDDV in all CPE positive cultures was confirmed by PCR and sequencing.
** Harvests of the cell passages of non-infected control sera pools were inoculated on fresh SK21 cells at 1:100 and 1:1000 dilutions for a second passage. Also in the second passage no CPE was observed.
