# Supplementary material for: A Novel Virus Causes Scale Drop Disease in Lates calcarifer
Source: PLoS Pathog. 2015 Aug 7;11(8):e1005074. doi: 10.1371/journal.ppat.1005074 (PMC4529248; doi:10.1371/journal.ppat.1005074)
Supplement: S5 Table — (PDF) [file ppat.1005074.s010.pdf]

S5 Table. Presence/absence of SDDV in serum of vaccinated fish on day 28

**Prototype Vaccines**	**Fish number**	**SDDV PCR**
Formalin-inactivated SDDV	1	**N***
	2	**N**
	3	**N**
	4	**N**
	5	**N**
Binary ethyleneimine-inactivated SDDV	1	**POS***
	2	**N**
	3	**POS**
	4	**POS**
	5	**N**
Recombinant Major Capsid Protein of SDDV	1	**N**
	2	**N**
	3	**N**
	4	**N**
	5	**N**
Placebo	1**	**POS**
	2	**POS**

* N: Negative, POS: positive in the SDDV PCR
** Results of the only two surviving fish
