# Supplementary material for: A Novel Virus Causes Scale Drop Disease in Lates calcarifer
Source: PLoS Pathog. 2015 Aug 7;11(8):e1005074. doi: 10.1371/journal.ppat.1005074 (PMC4529248; doi:10.1371/journal.ppat.1005074)
Supplement: S6 Table — (PDF) [file ppat.1005074.s011.pdf]

S6 Table. Primer and probe sequences used for SDDV qPCR and RSIV PCR

Primer	Sequence (5'-3')
SDDV-50-FW	CAG TGC ATT ACA AGA AAG
SDDV-143-PROBE	6FAM-ATG CCG TCA TTG TAA CAC TG-BHQ1
SDDV-213-REV	GCT GAA ACA ACA ATT TAG
RSIV IRIDO-FW-5	CGT GAG ACC GTG CGT AGT
RSIV IRIDO-REV-5	AGG GTG ACG GTC GAT ATG
